# Microbial Activities and Selection from Surface Ocean to Subseafloor on the Namibian Continental Shelf

**DOI:** 10.1128/aem.00216-22

**Published:** 2022-04-11

**Authors:** Aurèle Vuillemin, Ömer K. Coskun, William D. Orsi

**Affiliations:** a Department of Earth and Environmental Sciences, Paleontology & Geobiology, Ludwig-Maximilians-Universität München, Munich, Germany; b GeoBio-CenterLMU, Ludwig-Maximilians-Universität München, Munich, Germany; Georgia Institute of Technology

**Keywords:** *dsrA* and *aprA* genes, *Gammaproteobacteria*, stable isotope probing, *Bacteroidetes*, Benguela upwelling system, *Deltaproteobacteria* SAR324, *Gammaproteobacteria* SUP05, chemolithoautotrophy, cryptic sulfur cycle, dark carbon fixation, metatranscriptomics, oxygen minimum zones

## Abstract

Oxygen minimum zones (OMZs) are hot spots for redox-sensitive nitrogen transformations fueled by sinking organic matter. In comparison, the regulating role of sulfur-cycling microbes in marine OMZs, their impact on carbon cycling in pelagic and benthic habitats, and activities below the seafloor remain poorly understood. Using ^13^C DNA stable isotope probing (SIP) and metatranscriptomics, we explored microbial guilds involved in sulfur and carbon cycling from the ocean surface to the subseafloor on the Namibian shelf. There was a clear separation in microbial community structure across the seawater-seafloor boundary, which coincided with a 100-fold-increased concentration of microbial biomass and unique gene expression profiles of the benthic communities. ^13^C-labeled 16S rRNA genes in SIP experiments revealed carbon-assimilating taxa and their distribution across the sediment-water interface. Most of the transcriptionally active taxa among water column communities that assimilated ^13^C from diatom exopolysaccharides (mostly *Bacteroidetes*, *Actinobacteria*, *Alphaproteobacteria*, and *Planctomycetes*) also assimilated ^13^C-bicarbonate under anoxic conditions in sediment incubations. Moreover, many transcriptionally active taxa from the seafloor community (mostly sulfate-reducing Deltaproteobacteria and sulfide-oxidizing *Gammaproteobacteria*) that assimilated ^13^C-bicarbonate under sediment anoxic conditions also assimilated ^13^C from diatom exopolysaccharides in the surface ocean and OMZ waters. Despite strong selection at the sediment-water interface, many taxa related to either planktonic or benthic communities were found to be present at low abundance and actively assimilating carbon under both sediment and water column conditions. In austral winter, mixing of shelf waters reduces stratification and suspends sediments from the seafloor into the water column, potentially spreading metabolically versatile microbes across niches.

**IMPORTANCE** Microbial activities in oxygen minimum zones (OMZs) transform inorganic fixed nitrogen into greenhouse gases, impacting the Earth’s climate and nutrient equilibrium. Coastal OMZs are predicted to expand with global change and increase carbon sedimentation to the seafloor. However, the role of sulfur-cycling microbes in assimilating carbon in marine OMZs and related seabed habitats remain poorly understood. Using ^13^C DNA stable isotope probing and metatranscriptomics, we explore microbial guilds involved in sulfur and carbon cycling from ocean surface to subseafloor on the Namibian shelf. Despite strong selection and differential activities across the sediment-water interface, many active taxa were identified in both planktonic and benthic communities, either fixing inorganic carbon or assimilating organic carbon from algal biomass. Our data show that many planktonic and benthic microbes linked to the sulfur cycle can cross redox boundaries when mixing of the shelf waters reduces stratification and suspends seafloor sediment particles into the water column.

## INTRODUCTION

Oxygen minimum zones (OMZs) have long been recognized as hot spots for oxygen-sensitive nitrogen microbial transformations (1, 2) and are traditionally seen as regions dominated by heterotrophic denitrification and anaerobic ammonium oxidation (anammox) fueled by the sinking of organic matter (OM) produced via photosynthesis in the sunlit surface ocean (3–5). In regions such as the eastern tropical Pacific (6, 7), the Arabian Sea (8, 9), and the Benguela Current coastal upwelling (10), dropping oxygen concentrations in OMZ waters initiate a dynamic nitrogen cycle (11, 12), in which nitrate serves as the main terminal electron acceptor for the oxidation of OM and is successively converted to nitrite (13), nitrogen (N_2_), and nitrous oxide (N_2_O) gases through processes of denitrification (14) and autotrophic anammox (11). In general, anaerobic processes in the water column result in an overall geochemical deficit in inorganic fixed nitrogen relative to phosphorus (15, 16), and its loss from the oceans globally impacts the Earth climate system in terms of nutrient equilibrium and emissions of greenhouse gases (11, 17). Although the biogeochemistry and microbial ecology of oceanic OMZ regions were originally thought to fundamentally differ from those of euxinic basins (3, 18), phylogenetic surveys of sulfur-based microbial assemblages evidenced broad community similarities between oceanic OMZs and stratified basins (19, 20), questioning the factors driving diversification in sulfur-utilizing groups in analogously stratified systems (20).

In productive coastal OMZs, interactions between sulfide-rich sediments and the overlying water column lead to tightly coupled benthic-pelagic biogeochemical cycling (21–23), with blooms involving a succession of sulfide-oxidizing bacteria that detoxify sulfide (10, 24) and reduce nitrate to N_2_ while generating nitrite and ammonium (NH_4_^+^) that can augment nitrification and anammox (25, 26). However, direct evidence for large-scale active sulfur cycling in OMZs is still lacking, and the key sulfur and carbon metabolic processes catalyzed by OMZ microbial communities remain insufficiently characterized with regard to their taxonomic assemblages (12), population densities (27), gene content (28), and expression (29, 30). Oceanic oxygen levels are predicted to decrease significantly over the next decades in response to oceanic global change and human activities (31–33), triggering major shifts in nutrient cycling due to the expansion of OMZs (34). The resulting eutrophic conditions and overall increase in productivity are also expected to enhance particulate carbon sedimentation to the seafloor (35) and, thereby, benthic fluxes of N_2_O, hydrogen sulfide (H_2_S), and methane (CH_4_) mediated by the resident microbial communities (36, 37), and it is of the utmost importance to disentangle microbial processes coupling sulfur and carbon biogeochemical cycles within oceanic OMZ coastal waters and their underlying sediments.

The Benguela upwelling system (BUS) off Namibia is one well-studied area where nutrient-rich deep waters brought to the ocean sunlit surface highly stimulate primary productivity and result in naturally eutrophic conditions (38). As a result, oxygen is generally depleted below a 60-m water depth (mwd), with an OMZ reaching all the way down to the seafloor during austral summer (10, 39). In the summer months during periods of strong water column stratification, an absence of oxygen, as well as nitrate, in surface sediments on the shelf allows benthic H_2_S to diffuse upward and escape into the overlying OMZ and accumulate in the stagnant waters of the shelf (21, 39, 40). However, in the winter months the water column experiences increased mixing, and the reduced stratification causes oxygen and nitrate penetration to the seafloor, and benthic plumes of H_2_S into the water column are reduced (21, 39, 40). Thus, sulfur cycling in the OMZ of the Namibian shelf is expected to be strongly influenced by the activity of sulfur-oxidizing bacteria and seasonal oxygenation of the sediment-water interface (SWI) (41) that results from increased physical mixing in the winter and reduced stratification of the water column.

In austral winter 2018 (39, 40), an oceanographic sampling expedition to the BUS offered the opportunity to sample water column and sediment during the more well-mixed (and less stratified) conditions on the Namibian shelf for microbiology analyses. Here, we explore microbial activities, gene expression, and carbon assimilation in the water column and sediments of the Namibian shelf during winter conditions of increased mixing and reduced water column stratification. We detail metabolic guilds involved in sulfur cycling, their taxonomic compositions, and relative expression of functional marker genes, thereby providing new insights into coupled carbon and sulfur biogeochemical processes of a productive coastal OMZ during the winter months, which experience increased physical mixing and reduced water column stratification. Our data show that during this time, many microbial taxa from the benthic community are redistributed into the water column, where they continue to assimilate algal biomass, not only in the OMZ, but also in the surface ocean. Moreover, many of the microbial taxa from the water column community retain their metabolic activity after burial in the anoxic sediments, where they continue to express genes and assimilate carbon even into the sulfidic zone of the sediment column.

## RESULTS

### Water column and sediment geochemistry.

**FIG 1:**
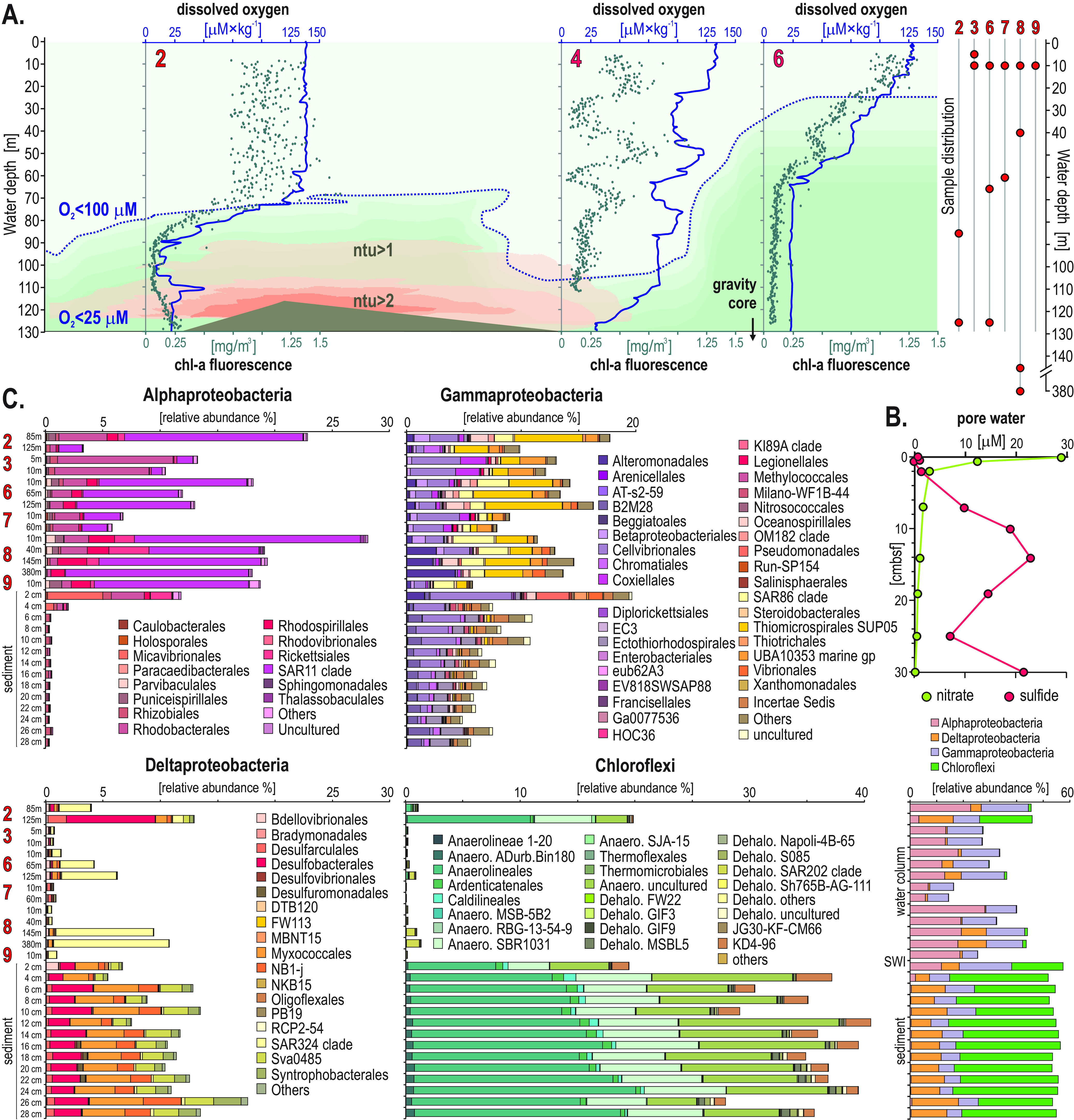
Profiles for dissolved oxygen, chlorophyll *a* (chl-*a*) fluorescence, and turbidity in Namibian shelf waters, sediment pore water profiles, and relative abundances of *Proteobacteria* and *Chloroflexi* and their taxonomic affiliations at the order level. (A) Vertical profiles of dissolved O_2_ in the water column at sampling sites 2, 4, and 6, which allowed definition of an oxycline (100 to 140 μM) and an OMZ (<60 μM) along the Namibian coast, and vertical profiles of chlorophyll *a* fluorescence [mg/m^3^] and turbidity in nephelometric turbidity units (ntu), which show the presence of mesoscale eddies in shelf waters and the suspension of seafloor sediments into the water column. (B) Geochemical profiles of pore water nitrate (green) and sulfide (red) showing a redox transition zone between 8 and 12 cmbsf, with relative abundances (%) of *Alpha*-, *Delta*-, and *Gammaproteobacteria* and *Chloroflexi* at different water depths at sites 2, 3, and 6 to 9 and in the upper 30 cm of sediment at site 6. (C) Relative abundances (%) of *Alpha*-, *Gamma*-, and Deltaproteobacteria and *Chloroflexi* at the order level at different water depths at sites 2, 3, and 6 to 9 and in the upper 30 cm of sediment at site 6.

**FIG 2:**
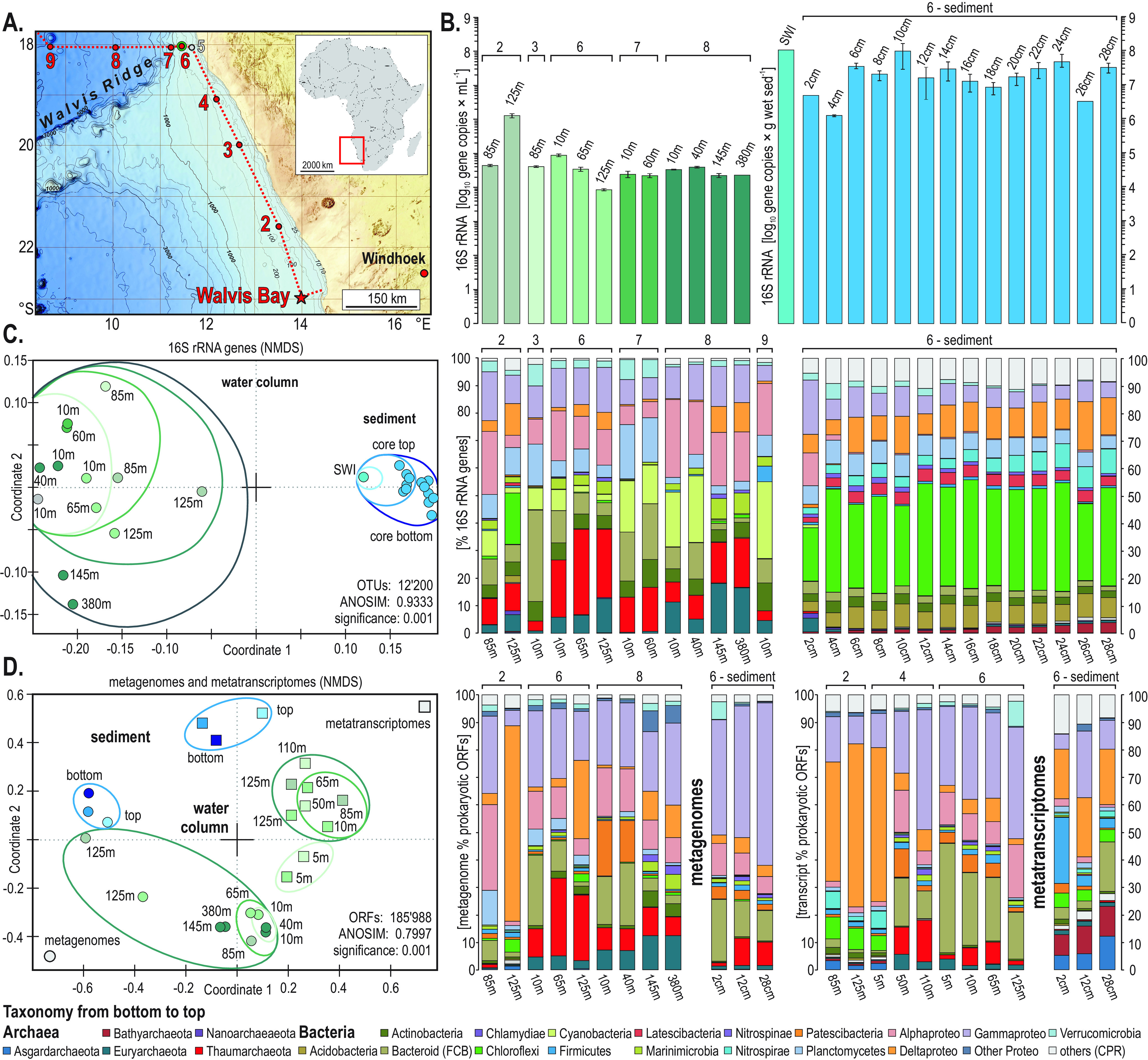
Sampling sites along the Namibian shelf, quantification of 16S rRNA genes, beta diversity, and taxonomic assemblages of 16S rRNA gene amplicons, metagenomes, and metatranscriptomes. (A) Bathymetric map of the Namibian shelf displaying the different sites sampled during the EreBUS cruise 2018. (Adapted from GEBCO Compilation Group [2021] GEBCO 2021 Grid [http://doi.org/10.5285/c6612cbe-50b3-0cff-e053-6c86abc09f8f]. (B) Quantitative PCR (qPCR) of total 16S rRNA genes in the water column (gene copies · mL seawater^−1^) and sediment (gene copies · g wet sed^−1^). (C) Nonmetric multidimensional scaling (NMDS) plot based on all OTUs across all water column and sediment samples (left) and their corresponding taxonomic assemblages (percent 16S rRNA genes). (D) NMDS plot based on all prokaryotic ORFs (left) from the metagenomes (circles) and metatranscriptomes (squares) and their corresponding taxonomic assignments (percent ORFs). SWI, sediment-water interface.

Consistent with prior studies (10, 11), an oxycline was observed between 50 and 95 mwd that exhibited O_2_ concentrations spanning 100 to 40 μM (Fig. 1A). Below 65 to 95 mwd, an OMZ defined as having O_2_ concentrations of <60 μM (36) was detected at all sites sampled along the shelf (Fig. 2A). Coastal bottom waters were not completely devoid of O_2_ (<25 μM) and were considered dysoxic (Fig. 1A; see Fig. S2 in the supplemental material). In the sediment underlying the OMZ waters at site 6, pore water chemical analyses indicated that nitrate and nitrite were consumed quickly at the sediment surface, followed by an accumulation of sulfide with depth (Fig. S2). Thus, the sediments exhibit a redox gradient spanning dysoxic (∼25 μM O_2_) conditions at the SWI, measurable nitrate down to 5 cm below the seafloor (cmbsf), and sulfidic conditions at 30 cmbsf (Fig. 1B). As reported previously, the sampled area of the OMZ in relatively shallow waters (100 to 125 mwd) was not receiving benthic H_2_S into the water column at the time of sampling—due to nitrate being abundant in the upper 10 cm of the sediment. The fluorescence profiles of chlorophyll *a* (chl-*a*) show maximum chl-*a* concentrations (i.e., 1.25 mg/m^3^) in the surface ocean at site 6 (Fig. S3), with a decreasing trend with water depth that runs in parallel to the oxygen profiles (Fig. 1A). Turbidity in the water column, measured as nephelometric turbidity units (ntu), is detectable (i.e., ntu >1) in the shelf bottom waters between site 2 and site 4 (Fig. S3). Together with the chl-*a* fluorescence profile at site 4, these data indicate the presence of water eddies along the continental shelf with suspension of seafloor sediments into the water column (Fig. 1A). This is typical of the winter months on the Namibian coast that has more well-mixed water conditions (21, 39, 40).

### Density, taxonomy, and beta diversity of 16S rRNA genes.

Across all sites sampled along the shelf (sites 2 to 6), microbial abundances based on quantitative PCR (qPCR) assays of 16S rRNA genes are about 10^5^ gene copies · mL^−1^ in the water column, increasing by 1.5 orders of magnitude in the OMZ waters at site 2 (Fig. 2A and B). Overall, microbial abundances tend to decrease northward along the coast. In comparison, the sites sampled further offshore (sites 7 and 8) display slightly lower and constant 16S rRNA gene densities (10^4^ gene copies · mL^−1^) compared to those on the shelf. In the sediment, concentrations of 16S rRNA genes are on the order of 10^8^ gene copies · g wet sediment (sed)^−1^, which is roughly 3 orders of magnitude higher than in the overlying water. Below the seafloor, the 16S rRNA gene profiles show that microbial biomass decreases by 2 orders of magnitude (10^8^ to 10^6^ gene copies · g wet sed^−1^) from the SWI to 5 cmbsf (Fig. 2B), which is consistent with the depletion of pore water nitrate (Fig. 1B, Fig. S2). Below 5 cmbsf, 16S rRNA gene densities increase and fluctuate around 10^7^ gene copies · g wet sed^−1^ as the sediment becomes sulfidic (Fig. 2B, Fig. S2).

In the water column, sequencing results of 16S rRNA genes show that the microbial assemblages mostly consist of *Cyanobacteria*, *Bacteroidetes* (FCB group, i.e., *Fibrobacteres*, *Chlorobi*, and *Bacteroidetes*), and *Alpha*- and *Gammaproteobacteria*. *Thaumarchaeota*, which represent 10% of the total microbial composition in surface waters at all sites, sum up to 30% of total 16S rRNA genes in the OMZ waters at site 6 (Fig. 2C). The 16S rRNA genes assigned to *Euryarchaeota* tend to increase from about 5% to 15% with water depth. The residual assemblages are mostly composed of *Actinobacteria*, *Bacteroidetes*, and Deltaproteobacteria. In the sediment, the 16S rRNA gene assemblage shows a clear predominance of *Chloroflexi*, complemented with mostly *Delta*- and *Gammaproteobacteria* (Fig. 2C). Contrasting with the water column, *Archaea* represent on average about 2% of the 16S rRNA gene sequences.

The nonmetric multidimensional scaling (NMDS) analysis based on 12,200 operational taxonomic units (OTUs) obtained from 16S rRNA gene sequencing clearly separates all water column and sediment samples (analysis of similarity [ANOSIM] *R* = 0.933, *P = *0.001). The resulting NMDS plot (Fig. 2C) evidences a significant shift in the community composition from surface waters (i.e., left end-member) across the OMZ waters down to the SWI and further into the sediment (i.e., right end-member).

Taxonomic assignments at the order level (Fig. 1C) indicate that the SAR11 (42), SUP05 (19, 43, 44), and SAR324 (45, 46) clades among *Alpha*-, *Gamma*- and Deltaproteobacteria, respectively, are predominant in the surface ocean and OMZ waters but nearly absent in the sediment. The SWI displays a clearly increased abundance of *Gammaproteobacteria*, such as *Halioglobus* (47), but overall, the relative abundance of *Gammaproteobacteria* decreases gradually with sediment depth. In the subseafloor, the *Myxococcales* with the Sva0485 clade among Deltaproteobacteria and *Anaerolineales* clades among *Chloroflexi* start ruling the microbial composition from 2 cmbsf and downcore (Fig. 1C).

### Taxonomy and beta diversity of metagenomes and metatranscriptomes.

**(i) Metagenomes.** Analysis of the metagenomes provided a way to cross-check whether taxonomic assignments based on PCR primers biased the relative abundance of taxa and vice-versa. Comparison between the two (Fig. 2C and D) shows that the archaeal distribution is similar across water column samples, whereas open reading frames (ORFs) assigned to *Proteobacteria* are somehow increased in the metagenomes. In the sediment, the number of ORFs assigned to *Gammaproteobacteria* apparently increases at the expense of *Chloroflexi*. Otherwise, the taxonomic assignments of metagenomes are consistent with those of 16S rRNA genes.

**(ii) Metatranscriptomes.** In the water samples, protein-encoding ORFs are by far often more assigned to Deltaproteobacteria along the seasonally sulfidic shelf waters (sites 2 and 4), whereas taxonomic assignments for ORFs from northern sampling sites mostly indicate *Bacteroidetes*, *Alpha*- and *Gammaproteobacteria*, and *Thaumarchaeota* as the main metabolically active phyla (Fig. 2D). In the sediment, *Firmicutes* and *Delta*- and *Gammaproteobacteria* represent about 50% of the expressed ORFs. Metabolic expression of ORFs by *Archaea* (i.e., *Bathyarchaeota*, *Asgardarchaeota*) increases to >20% with sediment depth.

The NMDS analysis based on all annotated protein-encoding ORFs clearly separates the metagenome and metatranscriptome samples (ANOSIM *R* = 0.799, *P = *0.001), significantly plotting samples at different positions spanning from the surface ocean across OMZ waters to sediment core top to bottom (Fig. 2D).

### Potential and expression of functional marker genes and carbon pathways.

**FIG 3:**
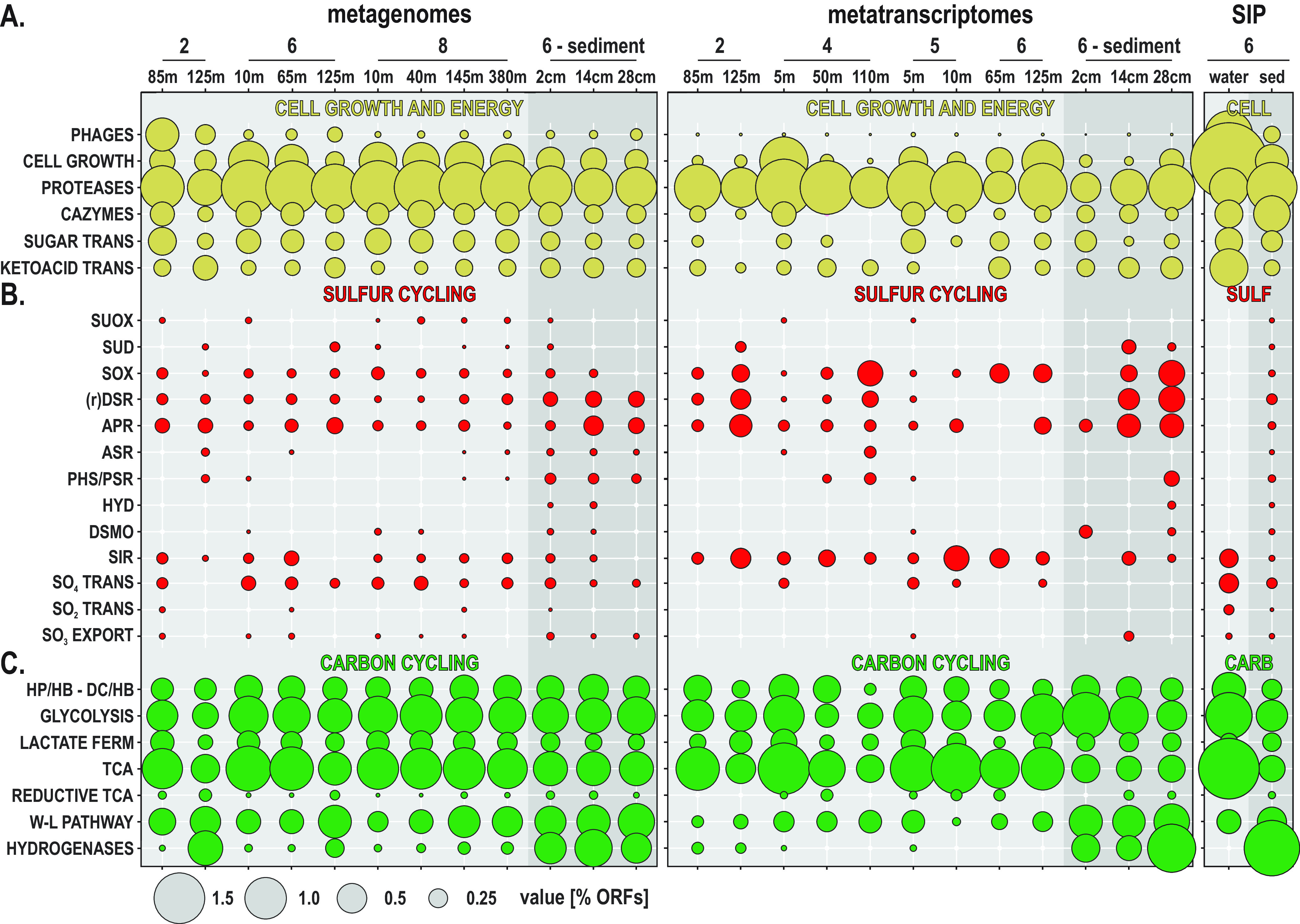
Metabolic potential and activities in the metagenomes, metatranscriptomes, and SIP incubations. (A to C) Bubble plot showing the relative abundances of metabolic functions (percentage of total ORFs) in the metagenomes, metatranscriptomes, stable isotope probing (SIP) water, and sediment incubations (left to right) assigned to (A) phages, cellular growth and energy, organic matter recycling, sugar and ketoacid transporters, and functional marker genes involved in (B) sulfur cycling and (C) carbon pathways. SUOX, sulfite oxidase; SUD, sulfide dehydrogenase; SOX, sulfur-oxidizing proteins; (r) DSR, dissimilatory sulfite reductase including reverse dissimilatory sulfite reductase; APR, adenylylsulfate reductase; ASR, anaerobic sulfite reductase; PHS/PSR, thiosulfate/polysulfide reductase; HYD, sulfhydrogenase; DSMO, dimethyl sulfoxide reductase; SIR, assimilatory sulfite reductase; HP/HB-DC/HB, 3-hydroxypropionate/4-hydroxybutyrate–dicarboxylate/4-hydroxybutyrate cycle; lactate ferm, lactate/pyruvate fermentation; TCA, tricarboxylic acid cycle; W-L pathway, Wood-Ljungdahl pathway.

In order to assess levels of metabolic expression per phylum, we proceeded to a comparison of the relative percentage of total prokaryotic ORFs identified in the metatranscriptomes to those present in the metagenomes. We focused on ORFs encoding genes involved in cell growth, OM remineralization, sulfur cycling, and heterotrophic and dark autotrophic carbon pathways (Fig. 3). Because we were interested in the coupling of sulfur cycling and dark carbon fixation within the OMZ, we looked for the ribulose-1,5-diphosphate carboxylase gene (*RuBisCO*) of the Calvin-Benson-Bassham (CBB) pathway (48) but excluded phototrophic microorganisms from the analysis (to focus on chemolithoautotrophic groups).

ORFs encoding biosynthesis of cell membrane, chromosome replication, and the ring divisome complex (ftsA-W) were used as indicators of transcriptional processes involved in cell growth. The highest numbers of expressed ORFs from this category are observed in surface waters at sites 4 and 5 and tend to decrease with water depth (Fig. 3A). In contrast, at site 6, the numbers of expressed ORFs encoding functions of cell growth and division increase downward and are maximal at 125 mwd in OMZ waters. In the sediment, the number of ORFs involved in cell growth increases downcore. Protease-related ORFs are generally more expressed than those of carbohydrate active enzymes (CAZymes) (49), with slightly higher relative percentages of expressed ORFs in surface waters (Fig. 3A). ORFs assigned to sugar transporters were significantly less expressed in OMZ waters than in the surface ocean, whereas transporters targeting ketoacids had a relatively high level of expression at the OMZ upper boundary (Fig. 3A).

**FIG 4:**
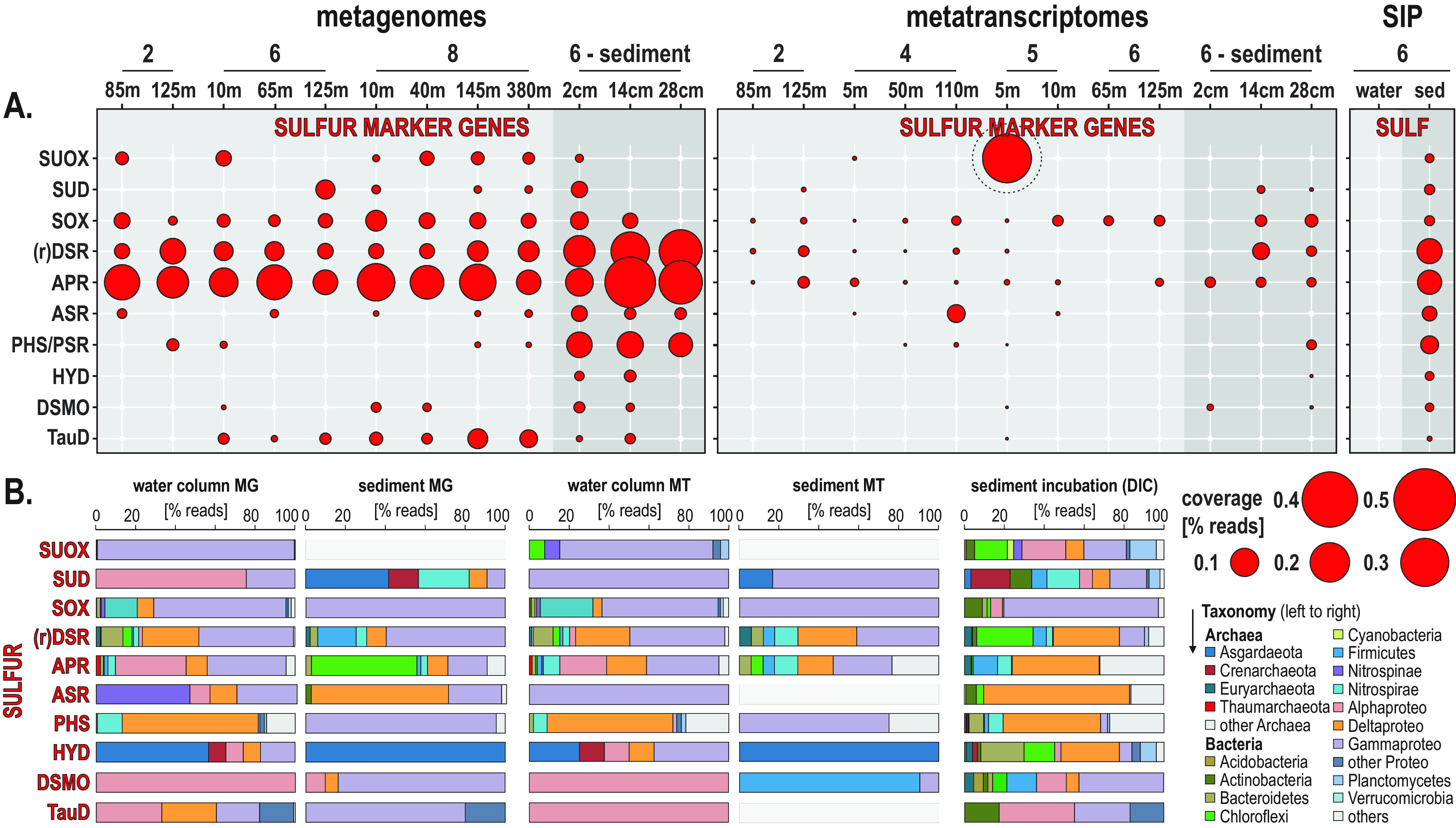
Metabolic functions and activities related to sulfur cycling in the water column, sediment, and SIP incubations and the corresponding taxonomic assignments at the phylum level. (A) Bubble plot showing the relative potential and expression of metabolic functions (percentage of total reads) assigned to sulfur cycling in the metagenomes, metatranscriptomes, and stable isotope probing (SIP) sediment incubations (left to right). (B) Taxonomic bar charts (percentage of reads) for the corresponding functional marker genes related to sulfur cycling at the phylum level in the metagenomes (MG), metatranscriptomes (MT), and anaerobic SIP incubations with sediment and ^13^C-labeled bicarbonate (DIC). SUOX, sulfite oxidase; SUD, sulfide dehydrogenase; SOX, sulfur-oxidizing proteins; (r) DSR, dissimilatory sulfite reductase and reverse dissimilatory sulfite reductase; APR, adenylylsulfate reductase; ASR, anaerobic sulfite reductase; PHS/PSR, thiosulfate/polysulfide reductase; HYD, sulfhydrogenase; DSMO, dimethyl sulfoxide reductase; TauD, taurine dioxygenase.

**FIG 5:**
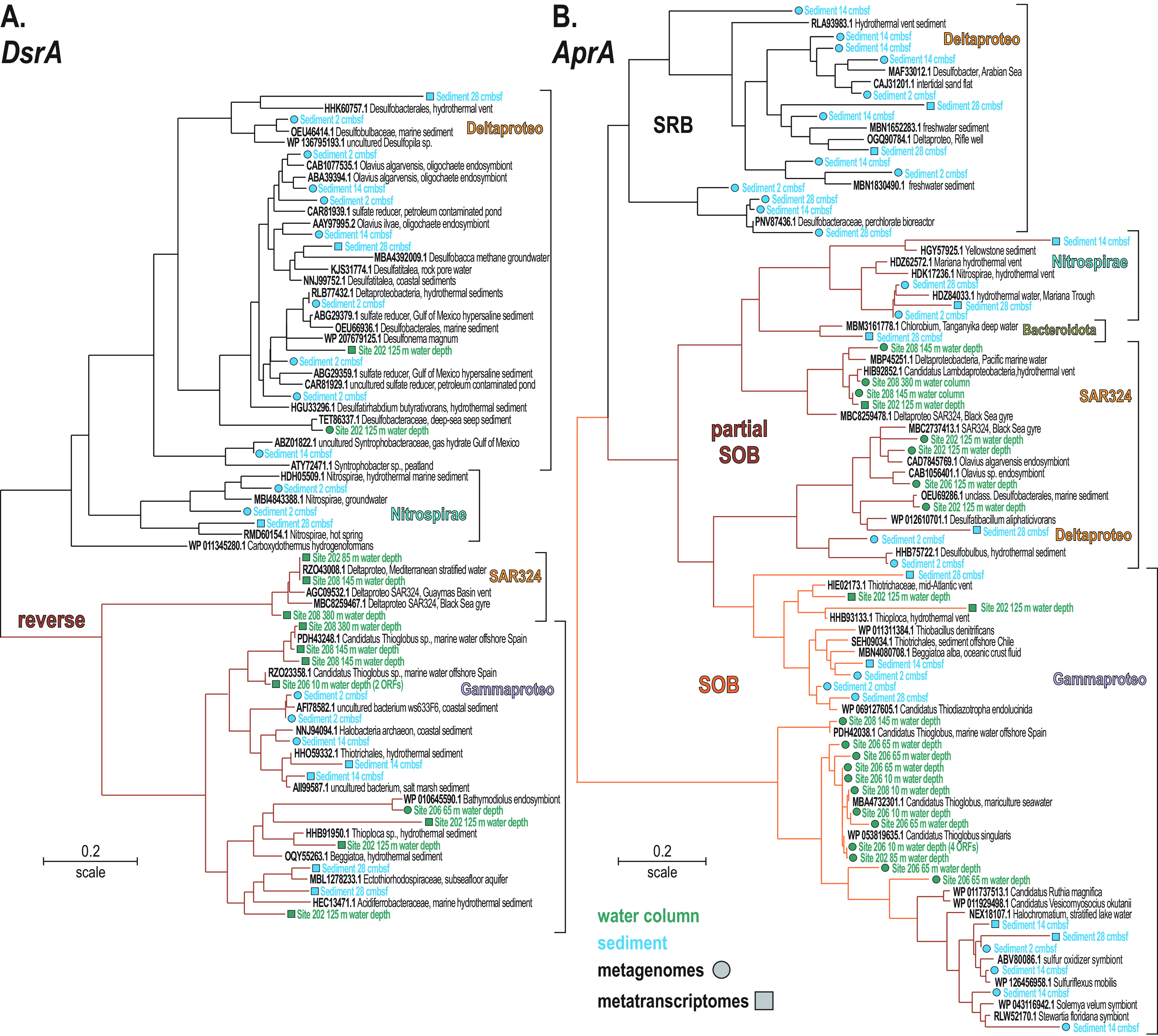
Phylogenetic analysis of predicted proteins encoded by selected marker genes in the metagenomes and metatranscriptomes, based on RAxML using BLOSUM62 as the evolutionary model. (A) Phylogenetic tree of all *dsrA* and *rDsrA* ORFs detected (466 aligned amino acid sites). (B) Phylogenetic tree of all *aprA* ORFs detected (754 aligned amino acid sites).

**(i) Sulfur.** To trace sulfur cycling microbial activities in the Namibian OMZ, we looked for the presence and expression of ORFs encoding genes with similarity to sulfite oxidase (*suox*), sulfide dehydrogenase (*sud*), periplasmic sulfur-oxidizing proteins (*sox*) as part of the sulfur oxidative pathway (50), dissimilatory sulfite reductase (*dsr*) and its reverse-acting homolog (*rDsr*), adenylyl-sulfate reductase (*apr*), anaerobic sulfite reductase (*asr*), thiosulfate/polysulfide reductase (*phs*/*psr*), sulfhydrogenase (*hyd*), dimethyl sulfoxide reductase (*dmso*), assimilatory sulfite reductase (*sir*) (51), taurine dioxygenase (*tauD*), and sulfate and sulfonate transporters as well as thiosulfate exporters. ORFs assigned to *sir* genes are expressed in the water column across all sites and relatively more abundant downward in the OMZ and with sediment depth (Fig. 3B), indicating active metabolic assimilation of sulfur. Expression of *suox* and bidirectional *sud* genes indicates ongoing but minimal aerobic sulfur oxidation in the water column and bottom core sediment. Expression of *rDsr* and *apr* genes was detected in OMZ waters along the shelf (sites 2, 4, and 5). When considering the expression of *dsr* and *rDsr* together, the highest levels were observed in the anoxic sulfidic sediment (Fig. 3B and 4A). However, almost all of the *dsr* genes were expressed exclusively in the sediment, whereas *rDsr* was found to be expressed in the water column and in the sediment (Fig. 5). Expression of ORFs related to sulfate transporters was only detectable in the surface ocean (Fig. 3B and 4A).

**(ii) Carbon.** To trace heterotrophic processes and energy production, we looked for ORFs related to glycolysis, the tricarboxylic acid (TCA) cycle, and lactate/pyruvate fermentation. All three heterotrophic processes are actively expressed across sampling sites and water and sediment depth, with generally higher levels of ORF expression involved in the TCA cycle and lactate/pyruvate fermentation in oxic and dysoxic waters, respectively (Fig. 3C and 5A). To trace autotrophic carbon fixation, we looked for ORFs specifically assigned to the 3-hydroxypropionate/4-hydroxybutyrate (HP/HB, aerobic) and dicarboxylate/4-hydroxybutyrate (DC/HB, anaerobic) cycles, the reductive TCA cycle, and the Wood-Ljungdahl (W-L) pathway. ORF expression related to HP/HB decreases with water depth, whereas ORFs related to the DC/HB anaerobic counterpart are expressed in deeper waters and sediment (Fig. 3C and 5A). Detection of ORFs expressing the reductive TCA cycle is low and limited to surface waters and bottom core sediment. ORF expression for the W-L pathway, inclusive of formate dehydrogenase (*fdh*), increases with water depth and is maximal in bottom core sediments (Fig. 3C and 5A). Expression of ORFs involved in production of fermentative hydrogen, such as the heterodisulfide reductase (*hdr*), methyl-viologen reductase (*mvh*), and coenzyme F420 hydrogenase (*frh*), drastically increases in the sediment (Fig. 3C, Fig. S4).

### Taxonomic assignment of functional marker genes and phylogenetic analysis.

To identify the main constituents of microbial guilds involved in sulfur and carbon cycling across the coastal OMZ, we used the relative coverage in percent total reads and class-level taxonomic affiliation of the ORFs corresponding to the aforementioned functional marker genes in the metagenomes, metatranscriptomes, and SIP metagenomes (Fig. 4 and 5) according to the isolation source (i.e., water column, sediment, and related SIP incubations).

**FIG 6:**
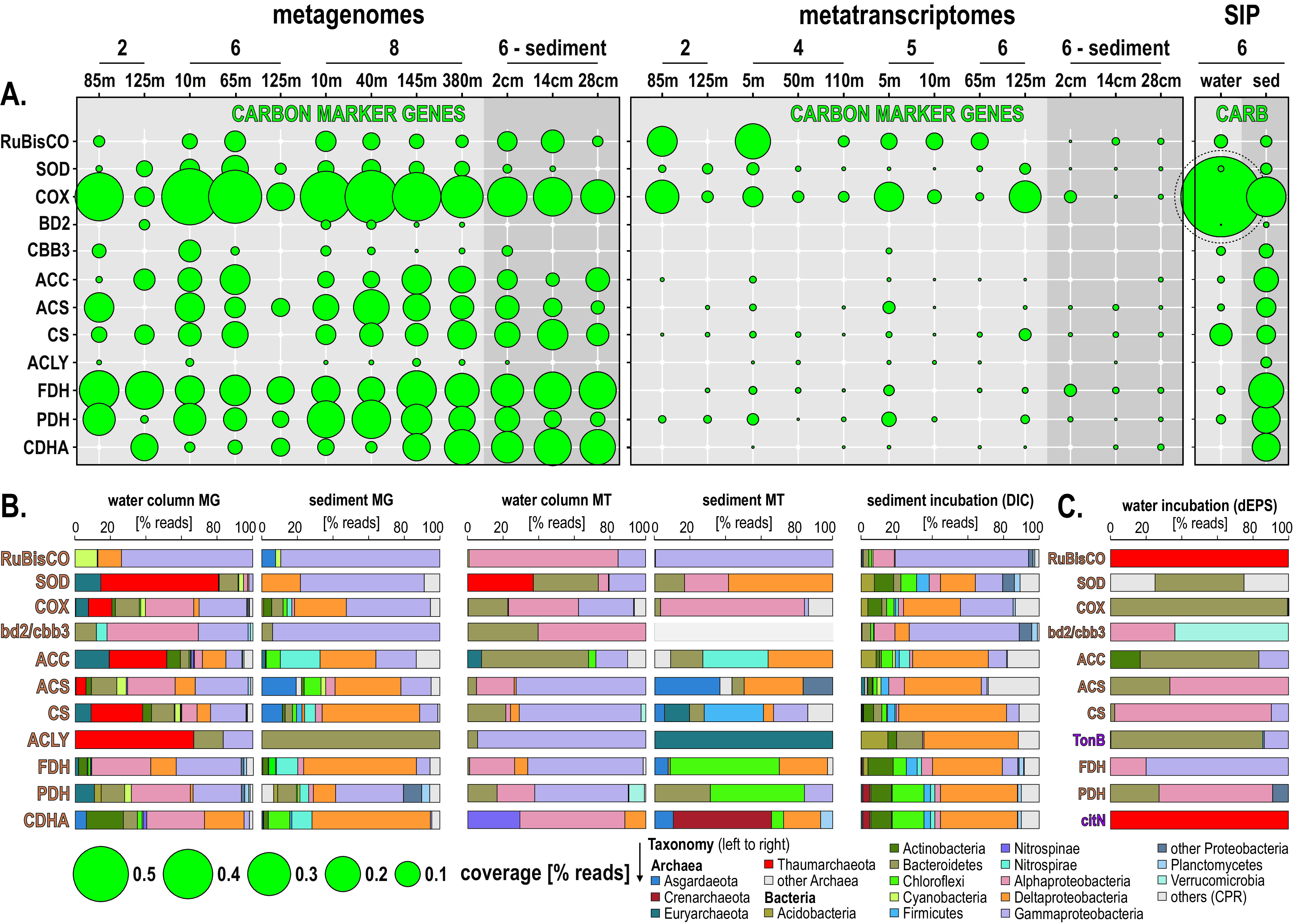
Metabolic functions and activities related to carbon cycling in the water column, sediment, and SIP incubations and the corresponding taxonomic assignments at the phylum level. (A) Bubble plot showing the relative potential and expression of metabolic functions (percentage of total reads) assigned to carbon cycling in the metagenomes, metatranscriptomes, stable isotope probing (SIP) water, and sediment incubations (left to right). (B) Taxonomic bar charts (percentage of reads) for the corresponding functional marker genes related to carbon cycling at the phylum level in the metagenomes (MG), metatranscriptomes (MT), and anaerobic SIP incubations with sediment and ^13^C-labeled bicarbonate (DIC). (C) Taxonomy of functional marker genes identified in aerobic incubations with water and ^13^C-labeled diatom mixture (dEPS). RuBisCO, ribulose-1,5-diphosphate oxidase; SOD, superoxide dismutase; COX, cytochrome *c* oxidase; bd2/cbb3, cytochrome *bd*_2_ ubiquinol and cytochrome *cbb*_3_-type oxidase; ACC, acetyl-coenzyme A carboxylase (i.e., HP/HB-DC/HP cycle); ACS, acetyl-coenzyme A synthetase (i.e., glycolysis); CS, citrate synthase (i.e., TCA cycle); ACLY, ATP-citrate lyase (i.e., reductive TCA cycle); FDH, formate dehydrogenase; PDH, pyruvate dehydrogenase (i.e., lactate/pyruvate fermentation); CDHA, carbon monoxide dehydrogenase to acetyl-coenzyme A synthase (i.e., Wood-Ljungdahl pathway); TonB, TonB-dependent (iron) transporters; citN, citrate transport protein.

Active expression of *sox* and *suox* genes was mostly affiliated with *Gammaproteobacteria* in the water column but was below detection in the sediment, whereas ORFs related to the *dsr*, *rDsr*, and *apr* genes were mostly expressed by *Delta*- and *Gammaproteobacteria*, some *Nitrospirae*, and a few *Firmicutes* in the water column and sediment (Fig. 4A and B). Although respiratory reduction of sulfur intermediates via *asr*, *phs*/*psr*, and *hyd* genes is apparently minor, the related expressed ORFs had highest similarity to organisms affiliated with *Delta*- and *Gammaproteobacteria* and *Lokiarchaeon* (Fig. 4B). Thus, expression of genes involved in sulfur oxidation (*suox*, *sox*, *rDsr*, *apr*) is ruled by *Gammaproteobacteria*, whereas Deltaproteobacteria and *Firmicutes* are the main phyla involved in sulfur reduction (Fig. 4A and B and 6). The relative coverage in percent total reads for genes encoding proteins involved in organic sulfur oxidation (*dsmo*, *tauD*) shows minor expression by *Alphaproteobacteria* (Fig. 4A and B).

To trace active microbial guilds involved in autotrophic and heterotrophic carbon cycling (Fig. 6), we used the following functional genes as markers: ribulose-1,5-diphosphate carboxylase gene (*RuBisCO*), superoxide dismutase (*sod*, i.e., H_2_O disproportionation), cytochrome *c* oxidase (*cox*, i.e., aerobic respiration), cytochrome *bd*_2_ ubiquinol (*bd2*) and cytochrome *cbb*_3_-type (*cbb3*) oxidases (i.e., microaerobic respiration) (52–54), acetyl-coenzyme A carboxylase (*acc*, i.e., HP/HB-DC/HP cycle), acetyl-coenzyme A synthetase (*acs*, i.e., glycolysis), citrate synthase (*cs*, i.e., TCA cycle), ATP-citrate lyase (*acly*, i.e., reductive TCA cycle), formate dehydrogenase (*fdh*, i.e., denitrification, acetogenesis), pyruvate dehydrogenase (*pdh*, i.e., lactate/pyruvate fermentation), and carbon monoxide dehydrogenase acetyl-coenzyme A decarboxylase/synthase (*codh/cdhA*, i.e., mostly W-L pathway).

In the water column, ORFs involved in aerobic metabolism (*sod*, *cox*) were mainly expressed by *Thaumarchaeota*, *Bacteroidetes*, and *Alpha*- and *Gammaproteobacteria*, whereas those involved in microaerobic metabolism (*bd2*/*cbb3*) were only expressed by *Alphaproteobacteria* (e.g., *Paracoccus*, *Thalassospira*), *Bacteroidetes*, and other FCB-related bacteria (e.g., *Pontibacter*, *Tenacibaculum*, *Imtechella*) (Fig. 6A and B). ORFs diagnostic of aerobic and facultatively anaerobic carbon fixation (*RuBisCO*, CBB pathway; *acc*, HP/HB cycle; *acly*, reductive TCA cycle) (55) were expressed by pelagic taxa among *Bacteroidetes* (56) and *Alpha*- and *Gammaproteobacteria* (Fig. 6B), with little detection of *Euryarchaeota* and *Chloroflexi*. Assignments of ORFs related to heterotrophic processes via glycolysis (*acs*) and TCA cycle (*cs*) indicate members of the *Gammaproteobacteria* and *Alphaproteobacteria* as the main actors of OM degradation in the water column. ORFs used as indicators of anaerobic fermentation (*pdh*) and the W-L pathway (*cdhA*) were mostly expressed by *Nitrospinae*, *Bacteroidetes*, and *Alpha*- and *Gammaproteobacteria* in the OMZ (Fig. 6A and B).

In the sediment, ORFs indicative of the CBB pathway (*RuBisCO*), microaerobic and anaerobic HP/HB cycle (*acc*), and reductive TCA (*acly*) were expressed by *Gammaproteobacteria*, *Nitrospirae*, and Deltaproteobacteria (Fig. 3B), respectively. Glycolysis (*acs*) and the TCA cycle (*cs*) were mostly expressed by *Euryarchaeota*, *Firmicutes*, and Deltaproteobacteria (Fig. 6B), whereas OM fermentation (*pdh*, *cdhA*) processes are apparently driven by *Chloroflexi* and *Bathyarchaeota* (Fig. 6B).

Our phylogenetic analysis of *dsrA* and *aprA* gene sequences was used to confirm and detail these results (Fig. 5). Transcripts identified from the water column as *rDsrA*, involved in sulfide oxidation (57), were assigned to the deltaproteobacterial clade SAR324 and *Gammaproteobacteria*, some of which were also present in the sediment (Fig. 5A). The *dsrA* gene sequences involved in dissimilatory sulfate reduction isolated from the sediment were all affiliated with Deltaproteobacteria and *Nitrospirae*, whereas only two *dsrA* sequences could be identified in the OMZ (water column) samples, closely affiliated with *Desulfonema* and *Desulfobacter* (Fig. 5A). In comparison, ORF sequences assigned to the *aprA* gene include sulfate-reducing, sulfur-oxidizing, and partial sulfur-oxidizing bacteria assigned to Deltaproteobacteria, *Gammaproteobacteria*, and *Nitrospirae* (Fig. 5B). Actively transcribed *aprA* gene sequences detected in both the water column and sediment are closely affiliated with some known cultivates, such as *Halochromatium*, *Beggiatoa*, *Thiotrix*, *Desulfatibacillum*, and *Desulfobacter*.

### Stable isotope probing incubations.

**FIG 7:**
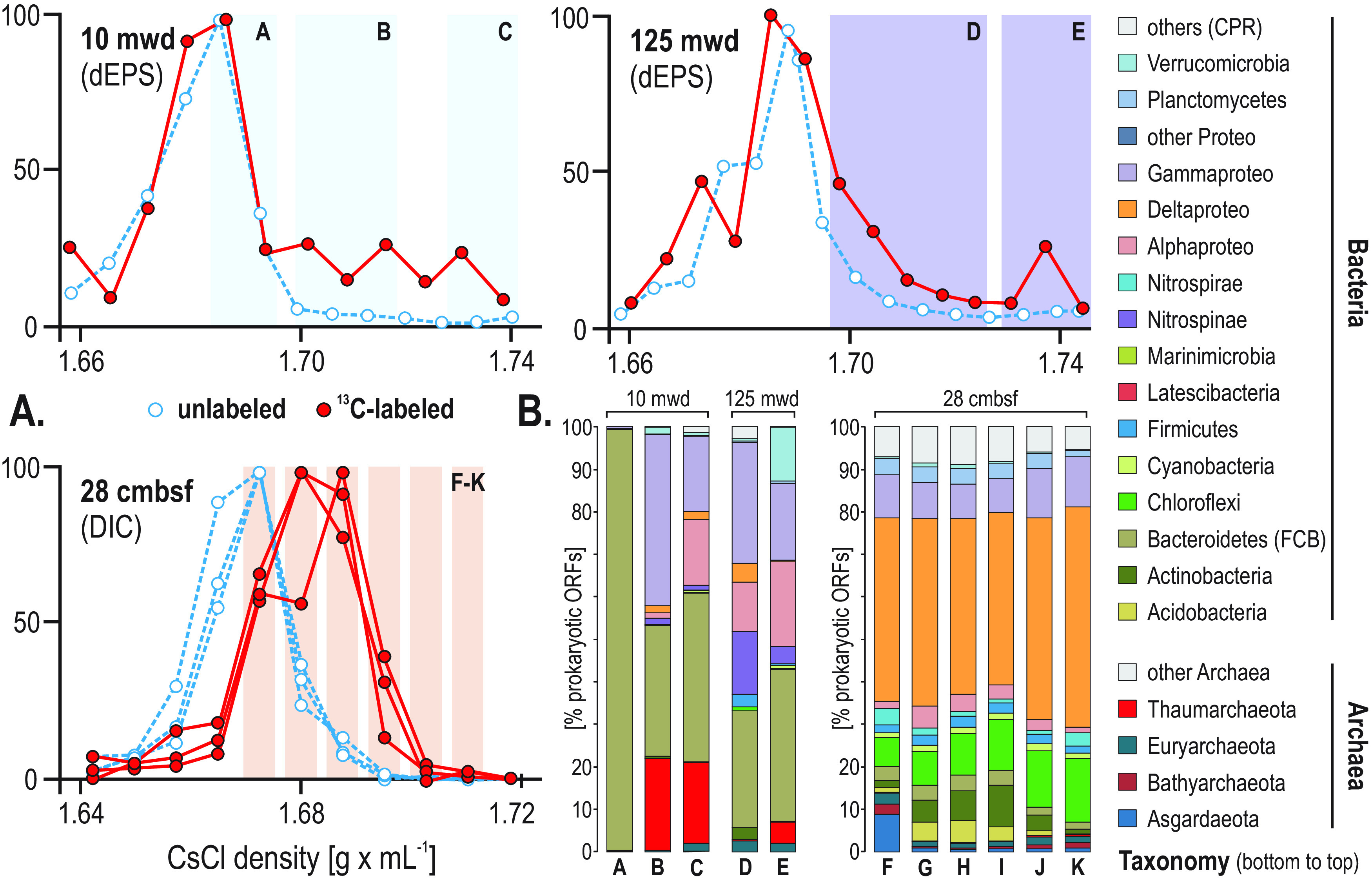
An increase in buoyant density of 16S rRNA genes in incubations with ^13^C-dEPS and ^13^C-bicarbonate and taxonomic assemblages from metagenomes prepared from “heavy” DNA fractions. (A) qPCR results of 16S rRNA genes for density fractions of DNA obtained from the 18- h SIP incubations with water from 10 and 125 mwd and ^13^C-dEPS and with sediment from 28 cmblf using ^13^C-bicarbonate at site 6 (red dots, ^13^C-labeled; blue dots, unlabeled controls). (B) Taxonomic assemblages of the “heavy” DNA fractions (percentage of prokaryotic ORFs) considered indicative of phyla showing ^13^C-dEPS and ^13^C-bicarbonate assimilation.

After 18 h of incubation in the dark at 10°C, the ^13^C-diatom exopolysaccharides (^13^C-dEPS) incubations showed ^13^C-labeling of 16S rRNA genes (defined by a shift in peak DNA buoyant density) in the surface ocean (10 mwd) and OMZ (125 mwd). Namely, “isotopically heavier” (i.e., ^13^C-enriched DNA) 16S rRNA genes compared to the unlabeled controls are indicated by the increased buoyant density of 16S rRNA genes in the ^13^C-incubations compared to controls that received the unlabeled substrate (Fig. 7). This shows that ^13^C-labeling of microbes synthesizing new DNA had occurred, which had assimilated the added ^13^C-dEPS into their biomass. Metagenomic sequencing of DNA from these heavy SIP fractions shows that most of the bacteria that had assimilated the added ^13^C-dEPS substrate are affiliated with *Bacteroidetes*, within both the 10-mwd and 125-mwd (OMZ) incubations (Fig. 7). Additionally, ORFs from heavy fractions affiliated with *Thaumarchaeota* increase in relative abundance at the highest CsCl densities, indicating that heterotrophic *Thaumarchaeota* (58) had also assimilated ^13^C-dEPS in the SIP incubations. ORFs encoding citrate transporters (*citN*) and *RuBisCO* affiliated with the *Thaumarchaeota* were detected in the heavy fractions from SIP incubations with ^13^C-dEPS (Fig. 6C, Fig. S5). In the oxic water SIP incubations with ^13^C-dEPS, ORFs from the ^13^C-labeled DNA fractions show that degradation and assimilation of algal matter proceed mostly via proteases and CAZymes involved in glycolysis and the TCA and HB/HP cycles (Fig. 3C). Phylogenetic analysis of ORFs encoding *sod*, *cox*, *acc*, and *acs* genes along with TonB-dependent transporters (*TonB*) confirms metabolic activities by *Bacteroidetes* in aerobic degradation of algal exopolysaccharide (EPS) and heterotrophic assimilation of carbohydrates via glycolysis (Fig. 6C, Fig. S5). Otherwise, ORFs related to aerobic glycolysis (*acs*) and the TCA cycle (*cs*) were mostly assigned to *Alphaproteobacteria* (Fig. 3C), which can apparently also evolve as microaerobes using cytochromes *bd*2 (54) and *cbb*3 (52) (e.g., *Paracoccus*, *Thalassospira*).

After 10 days of incubation in the dark at 10°C, the ^13^C-bicarbonate incubations amended with subseafloor sediment (59, 60) showed an increased buoyant density of 16S rRNA genes compared to the unlabeled controls, indicating that ^13^C-labeling of microbes synthesizing new DNA from bicarbonate via dark carbon fixation had also occurred in all incubations. These ^13^C-bicarbonate SIP incubations have been previously published and described elsewhere (59, 60). For our study here, we prepared new metagenomic libraries from the “isotopically heavy” DNA fractions and then sequenced and analyzed the data for functional marker genes (see highlighted regions of the CsCl gradient in Fig. 7). ORFs from the subseafloor ^13^C-labeled DNA fractions show that dark carbon fixation proceeds mostly via the W-L pathway and DC/HB in concomitance with acetogenic fermentation and production of molecular hydrogen (Fig. 3C). Proteases and CAZymes represent a high percentage of the detected ORFs involved in OM turnover in the sediment-heavy SIP metagenomes (Fig. 3A). ORFs involved in respiratory processes of sulfate reduction, sugar transporters, and ketoacid transporters were also detected in the sediment-heavy SIP metagenomes (Fig. 3A and B). Taxonomic affiliations of the ORFs in the sediment-heavy SIP metagenomes revealed ^13^C assimilation mainly by the *Delta*- and *Gammaproteobacteria*, *Chloroflexi*, and *Actinobacteria* (Fig. 7).

Taxonomic affiliations from the sediment-heavy SIP incubations of functional genes involved in sulfur cycling (Fig. 4) show that *Alpha*- and *Gammaproteobacteria* perform sulfur oxidation (*sox*, *suox*) and organic sulfur oxidation (*tauD*, *dsmo*), whereas Deltaproteobacteria and *Chloroflexi* can apparently be involved in mostly sulfate reduction (*dsr*, *apr*, *asr*, and *hyd*) but also sulfur oxidation (*suox*). ^13^C-labeled ORFs from the sediment-heavy SIP incubations encoding sulfide dehydrogenase (*sud*) further indicate that members of the *Bathyarchaeota*, *Actinobacteria*, *Nitrospirae,* and diverse *Proteobacteria* have metabolic capability to tolerate or possibly detoxify sulfide (Fig. 4B).

^13^C-labeled functional genes in the sediment-heavy SIP metagenomes for carbon assimilation (Fig. 6) evidence a majority of microaerobic (*bd2*/*cbb3*) and autotrophic (*RuBisCO*) Gammaproteobacteria on the one side, and heterotrophic (*acs*, *cs*), fermentative (*pdh*), and acetogenic (*cdhA*) Deltaproteobacteria, *Firmicutes*, and *Chloroflexi* on the other side. The ^13^C-labeling of some genes normally involved in autotrophic carbon fixation (*acc*, *acly*) had the highest similarity to taxa from the phyla *Acidobacteria* and Deltaproteobacteria. In contrast, *RuBisCO*, *acc*, and *acly* ORFs from the heavy SIP metagenomes in the sediment had similarity to taxa within the *Actinobacteria*, *Bacteroidetes*, *Nitrospirae*, and *Alpha*- and *Gammaproteobacteria* (Fig. 6B). For the sediment SIP incubations, we acknowledge that some of the ^13^C fixed from bicarbonate can be released as isotopically labeled metabolites, which are subsequently recycled and assimilated by heterotrophs via cross-feeding (61, 62) and during anaplerotic reactions (56, 63), i.e., nonautotrophic CO_2_ fixation expressed during TCA cycle-refilling reactions (55). The uncertainties associated with cross-feeding in the microbial food web increase as the incubation time increases (62, 64). Nevertheless, regardless of which pathway the ^13^C label took, its assimilation into microbial biomass is demonstrated here and shows that active microbial carbon assimilation took place, either as bicarbonate or released metabolites from autotrophic organisms.

**FIG 8:**
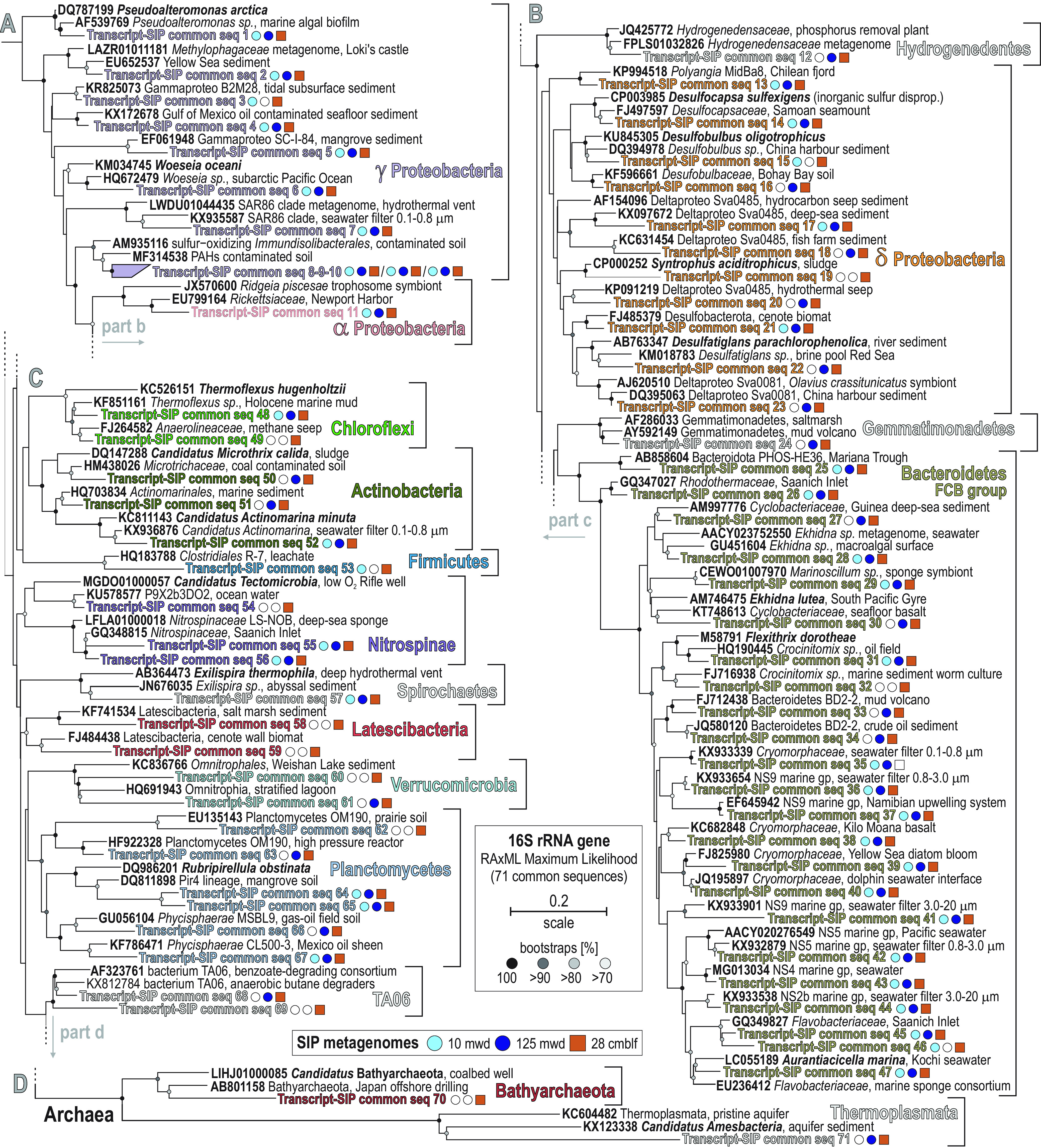
Phylogenetic analysis of 16S rRNA transcripts from metatranscriptomes and their detection as 16S rRNA genes within SIP metagenomes. RAxML maximum likelihood tree selected among 100 replicates for all partial 16S rRNA transcripts (V4 hypervariable region). The detection of these expressed transcripts as 16S rRNA genes within heavy metagenomes containing ^13^C-labeled DNA is displayed as symbols to the right of the sequence identifiers in the tree. The presence/absence of a 16S rRNA gene within a SIP metagenome (corresponding to expressed transcripts used to make the tree) is signified by full versus empty circles (10 and 125 mwd) and squares (28 cmblf), respectively. Bold type signifies accession numbers and cultivated species, whereas regular font indicates the sequence isolation sources.

We searched the metatranscriptomes for 16S rRNA transcripts that were also detected as 16S rRNA genes within the SIP metagenomes. This identified 16S rRNA transcripts from 71 taxa (Fig. 8) that had also assimilated either ^13^C-dEPS in the water column and/or ^13^C-bicarbonate in the sediments. In the sediments, these transcriptionally active and carbon-assimilating taxa are phylogenetically affiliated with taxa in the *Alpha*-, *Delta*-, and *Gammaproteobacteria* presumed to be involved in sulfur oxidation (*Woeseia*), disproportionation (*Desulfocapsa*), and reduction (*Desulfobulbus*, *Desulfatiglans*). Many 16S rRNA transcripts affiliated with the *Bacteroidetes* (FCB group), with closest matches related to aerobic heterotrophs isolated from seawater and algal blooms (*Ekhidna*, *Crocinitomix*, *Aurantiacicella*), assimilated ^13^C from dEPS in the surface ocean and OMZ but also assimilated carbon in the anoxic sediments (Fig. 8). Other 16S rRNA transcripts that were ^13^C-labeled correspond to aerobic to microaerobic heterotrophs from the *Planctomycetes* (*Rubripirellula*) and a few *Chloroflexi* (*Thermoflexus*). Similar to the *Bacteroidetes*, many of these taxa assimilated carbon in the sediments, OMZ, and surface ocean (Fig. 8). Many other 16S rRNA transcripts affiliated with the *Verrucomicrobia*, *Latescibacteria*, *Bathyarchaeota*, *Thermoplasmata*, and Deltaproteobacteria (*Syntrophus*) also assimilated carbon, but these showed more distinct patterns of carbon assimilation being either primarily within the sediments or in the water column (Fig. 8).

## DISCUSSION

### Population densities and taxonomic assemblies.

Our simultaneous characterization of taxonomic composition, metabolic gene content, gene expression, and carbon assimilation in the Namibian OMZ and its underlying sediment evidences a strong selection for distinct microbial communities in dysoxic waters and across the SWI (Fig. 1 and 2). On the shallow coastal shelf (site 2), 16S rRNA gene copy numbers increase over an order of magnitude from the surface down to the OMZ waters (i.e., 10^5^ to 10^6^ gene copies · mL^−1^), whereas elsewhere along the shelf and northward offshore (sites 3, 6, 7, and 8), gene densities slightly decrease with water depth (Fig. 2B). From the SWI into the surface sediment, abundances of 16S rRNA genes drop by 2 orders of magnitude (10^8^ to 10^6^ gene copies) within the upper 4 cmbsf, correlating with the depletion of pore water nitrate (Fig. 1B). As pore water sulfide builds up, gene densities increase (10^7^ to 10^8^ gene copies) and fluctuate in line with previously published qPCR values (i.e., 10^9^ to 10^7^) (27). The higher qPCR values, observed at 125 mwd at site 2, at the SWI, and at 10 cmbsf at site 6 (Fig. 2B) thus correspond with a transition to sulfidic conditions in bottom waters of the inner shelf and shallow sediment (Fig. 1), respectively (Fig. S2). The brief increases in gene copy numbers indicate that microbial populations are highly responsive to the availability of redox couples in their environment (65, 66). Microbial populations multiply faster at geochemical redox interfaces than under stable geochemical conditions (i.e., oxic, dysoxic, anoxic, sulfidic), as the sulfide diffusing upward can be oxidized with nitrate in surficial sediment and oxygen in bottom waters (Fig. 1A and B, Fig. S2).

Relative expression of ORFs indicative of cell growth and division (Fig. 3A) is maximal in surface oxic waters on the shelf, consistent with activities by primary producers and nitrifiers (3, 12, 67), but also increases in the OMZ distal waters northward (site 6). The taxonomic affiliation of 16S rRNA genes confirms previous surveys of the Namibian OMZ (3, 28) as it clearly separates two populations corresponding to the water column and sediment in the NMDS plot (Fig. 2C). The former is predominantly composed of diverse *Proteobacteria*, *Cyanobacteria*, *Bacteroidetes*, and *Thaumarchaeota*, whereas the latter consists mainly of *Chloroflexi*, *Nitrospirae*, and Deltaproteobacteria (Fig. 2C). *Chloroflexi* and *Nitrospirae* are already present at low abundances in the water column of the inner shelf OMZ, showing that the transition to anoxic and sulfidic conditions in the vicinity of the SWI and underlying sediment select for these two phyla (20, 68), whereas Deltaproteobacteria increase with sediment depth at the expense of *Alpha*- and *Gammaproteobacteria* (Fig. 1C and 2C). In spite of some taxonomic discrepancies, both NMDS analyses clearly separate samples from the surface ocean, OMZ waters, and sediment and confirm that the samples from the OMZ on the southern shelf (site 2, 125 mwd) are the most similar to sediment samples (Fig. 2C and D), thereby correlating with the presence of sulfidic redox interfaces (Fig. 1A and B). From the surface ocean down into sulfidic sediments, the geochemical gradient across the OMZ and SWI exerts strong selection on microbial assemblages (20, 68), with successively, *Cyanobacteria*, *Bacteroidetes*, *Alphaproteobacteria*, and *Planctomycetes* in surface waters, *Thaumarchaeota*, *Euryarchaeota*, and *Gammaproteobacteria* down into the OMZ, Deltaproteobacteria, *Nitrospirae* and *Chloroflexi* at the SWI, and specific archaea (e.g., *Bathyarchaeota*, *Lokiarchaeota*) further below (Fig. 2C).

The taxonomy at the order level (Fig. 1C) reveals the predominance of alphaproteobacterial SAR11 (42) and gammaproteobacterial SAR86 (69) clades in the surface ocean. These two cosmopolitan planktonic clades display genomic and metabolic streamlining with predicted advantages in dissolved OM assimilation (69). In OMZ waters, the gammaproteobacterial SUP05 (19, 44) and deltaproteobacterial SAR324 clades (45, 46) predominate in the taxonomic assemblages. These two clades display metabolic potential for sulfur oxidation via reverse *dsr* and *apr* genes (Fig. 5) with complementary metabolic capabilities to reduce nitrite (45) and consume ammonium under anaerobic conditions (43). The SWI shows an increase in nitrate-reducing *Gammaproteobacteria* (e.g., *Halioglobus*) (47), whereas the underlying sediment shelters a consortium of sulfate-reducing Deltaproteobacteria (e.g., *Desulfocapsa*, *Desulfobulbus*, *Desulfatiglans*, *Desulfomonile*, Sva0485 clade) and *Anaerolineales* clades among the *Chloroflexi* (Fig. 1C).

In contrast to northern distal waters (sites 4 and 6) where metabolic activities are dominated by *Thaumarchaeota*, *Bacteroidetes* and *Alpha*- and *Gammaproteobacteria*, the relative abundance of transcripts assigned to Deltaproteobacteria and *Chloroflexi* (Fig. 2D) shows that these phyla are among the most metabolically active in southern bottom waters of the inner shelf (site 2). In the sediment (site 6), *Firmicutes*, *Bathyarchaeota* (70), and *Lokiarchaeota* (60) are the most metabolically active phyla after the *Proteobacteria* and *Chloroflexi* (Fig. 2D). Altogether, the density and diversity of microbial populations across the different sites sampled along the Namibian coast (Fig. 1 and 2) suggest that variations in water column geochemistry (Fig. 1A) promote a succession of trophic interactions down into the sediment, and northward as lateral ventilation of bottom waters increases with mixing (10, 39). The geochemical pore water profiles are consistent with canonical reduction of nitrate and sulfate with sediment depth (Fig. 1B), whereas those of dissolved OM show that reduced forms of nitrogen and sulfur do not diffuse across the SWI back into bottom waters (Fig. S2).

### Aerobic and anaerobic sulfur cycling.

In the summer months, the SWI on the Namibian coastal inner shelf is anoxic and nitrate-depleted, and hydrogen sulfide and methane diffuse out of the sediment into the water column (10, 39, 40), triggering sulfur plumes (40). However, in the winter, the water column is less stratified due to increased mixing (Fig. 1A), with lateral ventilation of bottom waters that increases mixing toward the north (30). As a result of the increased mixing in winter, most of the H_2_S produced via sulfate reduction on the Namibian shelf is oxidized within the sediment before reaching the water column. This is attributed in part to diverse *Gammaproteobacteria* that couple sulfide oxidation with dissimilatory nitrate reduction (DNRA) using sulfur as an electron donor (10, 24, 71, 72). Consequently, anaerobic microorganisms performing sulfur transformations that favor DNRA to other nitrate-reducing pathways under sulfidic conditions are expected to be characteristic constituents of the microbial core of the Namibian OMZ (25, 72).

The number of expressed ORFs involved in *sox* proteins and bidirectional *sud* genes clearly increased with water depth into the OMZ and in the sediment, whereas those assigned to *suox* genes were not expressed under water dysoxic conditions (Fig. 3B). The same was observed for the ORFs expressing *dsr*, *rDsr*, and *apr* genes (57, 73), with levels of expressed ORFs increasing into the OMZ waters along the inner shelf but decreasing northward along the shelf. The relative abundance of expressed ORFs related to *sir* genes also increased in OMZ waters, demonstrating active metabolic assimilation of sulfur. Only a few ORFs involved in the respiration of sulfur intermediates (74), assigned to anaerobic *asr* and *phs/psr* genes, were expressed in the water column (Fig. 3B). The taxonomic assemblage of the water column metabolic guild actively expressing *dsr*, *rDsr*, and *apr* genes was in the majority composed of *Alpha*-, *Gamma*-, and Deltaproteobacteria, with few *Nitrospirae* and *Chloroflexi* (Fig. 4B); the predominance of gammaproteobacterial “*Thioglobus*” taxa (75) from the SUP05 clade (19, 44) and deltaproteobacterial SAR324 (45) clades evidence their important role in sulfur oxidation processes (Fig. 1C and 6). The potential for organic sulfur oxidation (*tauD*, *dsmo*) related to sinking OM (4, 76) was minor on the inner shelf (Fig. 4A) but increased in offshore waters (sites 7 to 9), and the expression of those genes was attributed mostly to the alphaproteobacterial SAR11 clade (Fig. 1C and 4B).

Phylogenetic analysis of the *rDsrA* genes evidenced the role of *Gammaproteobacteria*, such as “*Candidatus* Thioglobus” (SUP05 clade) (10, 44, 75), *Thioploca* (77, 78) and *Beggiatoa*, and the deltaproteobacterial clade SAR324 (Fig. 5A) as some of the main taxa driving sulfur oxidation in the water column (45). The taxonomic assignment of *aprA* genes was similar (Fig. 5B), albeit with a smaller number of expressed ORFs in the water column (Fig. 3B). The corresponding *aprA* gene-expressing taxa were closely assigned to *Thiothrix* (79), *Thioploca* (78, 80), and “*Candidatus* Lambdaproteobacteria” (81). These species presently found to actively express reverse *aprA* genes are known to store oxygen, nitrate, and elemental sulfur in vacuoles (78–80) and grow as filamentous colonies in both water and sediment. Our data indicate that they were mostly thriving at the SWI when nitrate and oxygen were available (Fig. 1A). In general, the expression of *dsrA* genes by sulfate-reducing bacteria was found to be limited in the water column (Fig. 3B and 4), in comparison to *rDsrA* genes, which were expressed in the water column and in the sediments (Fig. 5), indicating a separation of sulfate-reducing and sulfide-oxidizing habitats across the seawater-seafloor boundary.

In the sediment where reduction of sulfate and sulfur intermediates was a major process (60), canonical sulfate reduction in the sediment was actively dominated by Deltaproteobacteria (e.g., *Desulfobulbus*, *Desulfobacca*, *Desulfobacter*, *Desulfatitalea*, *Desulfonema*, *Desulfatirhabdium*) expressing the *dsrA* and *aprA* genes (Fig. 5A and B). These taxa are common representatives of sulfate-reducing consortia in marine settings (82, 83), some of which display metabolic capability to disproportionate elemental sulfur into reactive intermediate sulfur species (i.e., dismutation) (84, 85). Sulfur oxidation via *rDsrA* and *aprA* genes (57, 82) was also expressed in the sediment wherein it mostly relates to *Gamma*- and Deltaproteobacteria (e.g., *Beggiatoa*, *Sulfuriflexus*, *Desulfobulbus*) (Fig. 4B) as well as bacterial taxa affiliated with known symbionts (Fig. 5A and B) of oligochaete worms (86, 87) and bivalves (88), a macrofauna apparently adapted to Namibian sulfidic sediments (41). The expression of ORFs assigned to *phs*/*psr* genes indicated thiosulfate-related activity by *Gammaproteobacteria* (e.g., *Thiothrix*, *Ectothiorhodospira*). Consistently, profiles of dissolved organic matter (59) show that reduced forms of nitrogen and sulfur diffusing upward are oxidized in surface sediments and do not cross the SWI back into dysoxic bottom waters (Fig. S2). Interestingly in the bottom core, reduction of sulfur intermediates via *phs*/*psr*, and *hyd* genes (Fig. 3B and 4A) further involved *Lokiarchaeota* (60), *Bathyarchaeota* (74), and “*Candidatus* Muproteobacteria” (89). Anaerobic expression of *dsmo* genes included *Firmicutes* and *Gammaproteobacteria* (Fig. 4B).

In heavy SIP metagenomes from water column incubations with ^13^C-dEPS (Fig. 7), the ORF assignments did not evidence any dissimilatory sulfate reduction genes but only showed assimilatory sulfate reduction via *sir* genes with sulfate and sulfur oxide transporters (Fig. 3B and 4A). In contrast, heavy SIP metagenomes from sediment with ^13^C-bicarbonate resulted in ^13^C-labeling of all the genes successively involved in respiratory processes of sulfate reduction (Fig. 3B and 4A), which implies active carbon assimilation by sulfate-reducing bacteria (Fig. 6). The phylogenetic tree for 16S rRNA transcripts from metatranscriptomes, which were also detected as 16S rRNA genes within sediment SIP metagenomes (Fig. 8), confirms the presence of a highly active consortium of sulfur-oxidizing and sulfate-reducing taxa among the *Alpha*-, *Gamma*-, and Deltaproteobacteria (e.g., *Woeseia*, *Desulfocapsa*, *Desulfobulbus*, *Desulfatiglans*) with several *Chloroflexi* and *Firmicutes*, respectively.

Based on the expression profiles of *dsr*, *apr*, *asr*, and *phs*/*psr* genes (Fig. 4A), a cryptic sulfur cycle driven by dissimilatory sulfate reduction off the Namibian shelf waters was considered unlikely during austral winter. Although the related ORF coverage in the metagenomes increased offshore (Fig. 4A), transcript data from offshore sampling sites are lacking to show that such a cryptic sulfur cycle can be decoupled from benthic processes and lateral transport (25, 39, 40). Instead, the data show high activity by sulfur and sulfide oxidizing *Gammaproteobacteria* (SUP05 clade) and Deltaproteobacteria (SAR324 clade) (e.g., expressing the *rDsr* and reverse *apr* genes) and support prior studies of the Namibian (24, 25), Arabian (9, 90), and Peruvian (23, 71) OMZs that reported sulfide oxidation in the water column as a key process tightly coupled with benthic fluxes (10). Although not precluding a cryptic sulfur cycle, the deltaproteobacterial SAR324 clade (46), which was found most abundantly in deep offshore waters (Fig. 1C), only expressed r*DsrA* and *aprA* genes (Fig. 5). The present gene expression profiles further support that sulfate reduction in the water column represents a minor source of sulfide compared to the sediment and that planktonic sulfide-oxidizing bacteria are mainly sustained from benthic fluxes. Our data further identify the canonical consortium of sulfate-reducing bacteria in the sediment that is responsible for the production and releases of the H_2_S gas and the associated sulfur-oxidizing bacteria present as filamentous bacteria (79, 80). In addition, the identification of *dsrA*, *rDsrA*, and *aprA* gene expression by novel clades affiliated with the Deltaproteobacteria SAR324 clade, *Gammaproteobacteria*, and *Nitrospirae* (Fig. 5) widens the diversity of species involved in sulfur transformation in OMZs and the underlying sulfidic sediment.

### Carbon-assimilating microbes in the water column and sediments.

Expression of marker genes for aerobic respiration, such as the *sod* and *cox* genes, provided evidence for *Thaumarchaeota*, *Bacteroidetes* and *Alpha*- and *Gammaproteobacteria* as the main active aerobes in the water column (Fig. 6B), whereas gene expression for microaerobic respiration (*bd2*/*cbb3*) (52, 53) was low, involving mostly *Bacteroidetes* and other FCB-affiliated bacteria (e.g., *Pontibacter*, *Tenacibaculum*, *Imtechella*) and *Alphaproteobacteria* (e.g., *Paracoccus*, *Thalassospira*) (Fig. 6B). Autotrophic processes along the coastal OMZ related to the CBB and HP/HB cycles and the reductive TCA and W-L pathways (91, 92) inferred from *RuBisCO*, *acc*, *acly*, and *cdhA* gene coverage (Fig. 6A) were found to be mostly expressed by *Bacteroidetes*, *Alphaproteobacteria*, *Gammaproteobacteria*, and *Nitrospinae*, respectively (Fig. 6B). In the surface ocean, *RuBisCO* genes were assigned to specific nitrifying and sulfur-oxidizing *Alpha*- and *Gammaproteobacteria* (e.g., *Nitrobacter*, *Starkeya*, *Nitrosomonas*) (Fig. 6B). In OMZ waters, ORFs related to *acly* genes, which is the first step of the reductive TCA cycle (93), were expressed by *Gammaproteobacteria* and *Bacteroidetes*. In addition, down into the OMZ, ORFs with similarity to *fdh* genes were increasingly expressed by *Alpha*-, *Gamma*-, and Deltaproteobacteria (Fig. 6A), with formate used as an electron donor during denitrification (94) or as a carbon source in the first step of acetogenesis (95). Expressed ORFs encoding genes with similarity to proteins involved in glycolysis and the TCA cycle had the highest expression levels in the water column and tended to decrease under OMZ conditions (Fig. 3C and 5A). Taxonomic assignments of the *acs*, *cs*, and *pdh* genes (Fig. 6B) further indicated that the most active heterotrophs in the water column were related to *Bacteroidetes* and *Alpha*- and *Gammaproteobacteria*. The significant presence and activities of *Bacteroidetes* in the water column (Fig. 2C and D) suggest that they may be responsible for the turnover of sinking OM (56, 63), consuming oxygen in the surface ocean and, thereby, initiating microoxic conditions in the OMZ, wherein they can apparently shift to microaerobic respiration and reductive TCA cycle (Fig. 6B).

This was consistent with the heavy SIP metagenomes derived from seawater incubations with ^13^C-dEPS, which showed ^13^C assimilation from taxa affiliated with the *Bacteroidetes*, *Thaumarchaeota*, and *Alpha*- and *Gammaproteobacteria* (Fig. 7 and 8). Altogether, the high diversity of 16S rRNA transcripts expressed by FCB-affiliated bacteria, and the ^13^C-labeling of the 16S rRNA genes from these same OTUs in ^13^C-dEPS SIP incubations (Fig. 7 and 8), is consistent with the studies showing that the FCB-affiliated bacteria assimilate algal polysaccharides (96, 97). Relatively faster growth of *Bacteroidetes* feeding on algal biomass could lead to microoxic conditions (98) under which anaerobic respiration and fermentation processes are initiated in OMZ waters.

Although many phyla displayed metabolic potential for driving the autotrophic HP/HB-DC/HP cycle, ORF expression for *acc* genes was almost exclusively assigned to *Bacteroidetes* (63, 98, 99) in the water column and corresponding SIP incubations (Fig. 6A and B). The assignment of *acs*, *cs*, *pdh*, and cytochromes *bd*2 and *cbb*3 genes to *Alphaproteobacteria* indicated heterotrophic processes of dEPS assimilation via microaerobic glycolysis, whereas the *acc* and *cs* genes assigned to *Gammaproteobacteria* pointed to uptake of ^13^C-labeled CO_2_ produced during fermentation (i.e., anaplerosis) (55, 64). Although *Thaumarchaeota* are strongly selected against under anoxic conditions (100), they were labeled in both dEPS incubations with water samples from 10 and 125 mwd (OMZ) (Fig. 7). Interestingly, *Thaumarchaeota*, in spite of their metabolic potential for autotrophic carbon fixation (101), did not express *acc* or *acly* genes in the ^13^C-dEPS SIP metagenomes (Fig. 6C). Instead, they apparently expressed *RuBisCO* genes for inorganic carbon fixation (Fig. 6B). In addition, several ^13^C-labeled ORFs encoding citrate transporters (*citN*) affiliated with *Thaumarchaeota* (Fig. 6C, Fig. S5) indicate that *Thaumarchaeota* may contribute indirectly to dEPS cycling by assimilation of citrate, which could further explain their current ^13^C-labeling in water SIP metagenomes (Fig. 7). This also suggests citrate as a possible intermediate in the microbial degradation of dEPS within marine OMZs (102), with marine *Thaumarchaeota* being a potential key assimilator of enzymatically degraded algal EPS. These results underscore previous results highlighting the importance of particular planktonic heterotrophic *Thaumarchaeota* for the marine carbon cycle (58).

In the sediment, inorganic carbon fixation could proceed via multiple pathways (Fig. 6A) (91, 92). *RuBisCO* genes assigned to sulfur-oxidizing *Gammaproteobacteria* (e.g., *Thiothrix*, *Sulfuricella*, *Methylophaga*) were increasingly expressed below the SWI, evidencing their contribution to dark CO_2_ fixation via the CBB pathway (19, 43, 71, 103). Expression for the microaerobic to anaerobic DC/HP cycle via *acc* genes (55) mostly designated *Bacteroidetes*, *Nitrospirae* (104), and Deltaproteobacteria (Fig. 6B), whereas *acly* genes were expressed by *Euryarchaeota*. In addition, acetogens that couple anaerobic glycolysis with the W-L pathway require fermentative H_2_. Hydrogenase activities responsible for H_2_ production drastically increase in the seafloor (Fig. 3C) and mostly relate to heterodisulfide reductase (*hdr*), methyl-viologen reductase (*mvh*), and coenzyme F420 hydrogenase (*frh*) (Fig. S4). Their taxonomic assignments correlated with those of the *fdh* and *cdhA* genes (Fig. 6B) that were mostly expressed by *Lokiarchaeota* (38), *Bathyarchaeota* (70), *Chloroflexi*, (92) and Deltaproteobacteria, thereby confirming highly active acetogenic fermentation via the W-L pathway in the sediment. In terms of heterotrophic processes, expressed ORFs assigned to glycolysis (*acs*), the TCA cycle (*cs*), and fermentation (*pdh*) pointed to *Bacteroidetes*, *Chloroflexi*, *Firmicutes*, *Nitrospirae*, and Deltaproteobacteria as the most active heterotrophs in the sediment. Concomitant expression of the *acc*, *acly*, and *cdhA* genes by these phyla suggest that, in addition to chemolithoautotrophy by *Gammaproteobacteria* (82, 103), heterotrophic CO_2_ fixation may be significant in the sediment (105).

In SIP metagenomes from sediment incubated with bicarbonate, the ^13^C-labeled phyla that were most enriched were *Delta*- and *Gammaproteobacteria*, *Chloroflexi*, *Bacteroidetes*, and *Actinobacteria* (Fig. 7). These results could confirm inorganic carbon fixation by autotrophic *Gammaproteobacteria* (e.g., *Thiothrix*, *Thiocystis*, *Thiorhodococcus*, *Thiobacillus*, *Methylophaga*) via *RuBisCO* genes (103), and by heterotrophic Deltaproteobacteria, *Chloroflexi*, *Nitrospirae* (104), *Bacteroidetes*, *Actinobacteria*, and *Bathyarchaeota* via *acc*, *acly*, and *cdhA* concomitant with *acs*, *cs*, and *pdh* genes (Fig. 6B). In addition to SIP-labeled 16S rRNA genes of the ^13^C-bicarbonate incubations, the 16S rRNA transcripts corresponding to *Lokiarchaeota* (60), *Bathyarchaeota* (70), and *Chloroflexi* (106) were only detected in the inner shelf OMZ and subseafloor sediments, showing that their activities are limited to anoxic and sulfidic conditions (Fig. 7 and 8). These results are consistent with prior studies suggesting that these phyla are (homo)acetogens with metabolic capability to reduce sulfur and adapt to sulfidic conditions (60, 107). Several other OM fermenters (e.g., *Planctomycetes*, *Latescibacteria*, *Verrucomicrobia*) grew in SIP incubations amended with ^13^C-labeled bicarbonate (Fig. 7 and 8), suggesting heterotrophic inorganic carbon fixation (64) possibly during cross-feeding (55, 62). Many of the FCB-affiliated 16S rRNA transcripts that were detected in the water column and below the seafloor also became ^13^C-labeled under both oxic and anoxic conditions with ^13^C-dEPS and ^13^C-bicarbonate, respectively (Fig. 7 and 8). Therefore, it appears that many of the same *Bacteroidetes* taxa play important roles in carbon cycling in the seawater and in the sediments, which are potentially transported from the seafloor back into the water column during seasonal mixing (Fig. 1A) and carried offshore suspended in eddies and filaments (108, 109).

**Conclusions.** Our study shows that while strong selection and differential activities were observed across the SWI, a relatively large number of taxa from the seawater microbial community continue to actively assimilate carbon after being buried in the anoxic sediments. Conversely, many taxa adapted to nutrient conditions in the sediment also actively assimilate organic carbon under OMZ conditions, such as those reproduced in our SIP incubations. Our data show that many of these microbes are also linked to the sulfur cycle. Several heterotrophic microbes apparently derived from benthic communities display metabolic versatility in carbon uptake across different geochemical niches and could thereby actively assimilate algal biomass in the OMZ and also in the surface ocean. While the activity of water column-derived microbes in the sediments can be explained by their transport via sinking particles, the latter case (sediment microbes assimilating algal biomass in the surface ocean) seems a bit counterintuitive. However, we infer that this could result from seasonal suspension of seafloor sediments during increased mixing of bottom waters, which form part of the extensive nepheloid layer whereby massive lateral sediment transport occurs on the Namibian shelf (108–110). The importance of sediment suspension in spreading metabolically versatile microbes for planktonic carbon assimilation processes could be assessed in other settings to confirm whether this is a more widespread phenomenon in continental shelf environments or is specific to the Namibian shelf.

## MATERIALS AND METHODS

### Sampling.

The research expedition Meteor M148-2 entitled EreBUS (i.e., processes controlling the emissions of greenhouse gases from the Benguela upwelling system) took place in 2018 from 2 to 20 July and was a transit from Walvis Bay, Namibia, to Las Palmas, Canary Islands (Fig. 2A). The water column and underlying sediments were sampled at multiple sites on the shelf in a south-north transect and offshore as the ship transited northward. Full details of sampling can be found in a previously published paper from this same expedition (59), and a map of sample locations can be found in Fig. 2. Water and sediment samples retrieved from the Namibian continental shelf (18.0°S, 11.3°E) were taken on board the *F/S Meteor* vessel and directly frozen during the expedition (59, 60). The depth of the water column ranged from 100 to 125 m on the Namibian continental shelf.

**(i) Water column.** Sampling for the water column was described previously in reference 59. In brief, water samples were retrieved using a Niskin rosette equipped with a conductivity-temperature-depth system (10). At each site, 2 L of seawater was filtered, spanning 125 mwd on the shelf to 380 mwd further from shore (Fig. 2A). Water was sampled directly from the Niskin rosette into acid-washed 2-L glass flasks and filtered immediately via peristaltic pumping onto an in-line 0.2-μm polycarbonate filter (59). Replicate filters for DNA and RNA analyses were conditioned and stored in sterile DNA/RNA clean 15-mL Falcon tubes and frozen immediately at −80°C.

**(ii) Sediment cores.** Sampling for the water column was described previously in reference 59. In brief, a 30-cm-long sediment core was retrieved from 125 mwd at site 6 (Fig. 2A) using a multicorer, which yielded an intact SWI and the upper 30 cm of underlying sediment (Fig. S1). Immediately after retrieval, one core was processed at 4°C and sectioned into 2-cm intervals (60, 111). Sections were transferred immediately into sterile DNA/RNA-free 50- mL Falcon tubes and frozen immediately at −80°C until DNA and RNA extractions.

### Nucleic acid extractions.

Nucleic acid extractions were described previously (59). In brief, DNA was extracted from the filters following a published protocol (112). In brief, 850 mL of a sucrose EDTA lysis buffer (0.75 M sucrose, 0.05 M Tris-Base, 0.02 M EDTA, 0.4 M NaCl, pH 9.0) and 100 mL of 10% sodium dodecyl sulfate were UV-treated for 30 min and added to 2-mL gasketed tubes containing the filters and 0.1-mm sterile glass beads. Bead beating was performed for 1 min at 6 m · sec^−1^, and the samples were subsequently heated at 99°C for 2 min. After heating, 25 mL of 20 mg · mL^−1^ proteinase K was added, and samples were incubated at 55°C overnight. DNA was extracted and purified from the lysate using the DNeasy blood and tissue kit (Qiagen). The DNA from the sediments was extracted from 1 g using a sodium phosphate buffer and concentrated into 50-kDa Amicon filters, as described in a previous publication (60). DNA concentrations were quantified using a Qubit 3.0 fluorometer (Thermo Fisher Scientific).

In brief, RNA was extracted from either 0.5 g of sediment or from filters, using the FastRNA Pro Soil-Direct kit (MP Biomedicals) following the manufacturer’s instructions, with final elution of templates in 40 μL PCR water (Roche) with some modifications to maximize RNA yield and reduce DNA contamination as described previously (106, 113). In order to maximize recovery of the RNA pellet, we added 4 μL glycogen at 1 μg · mL^−1^ prior to the 30-min isopropanol precipitation.

The ^13^C-labeled diatom exopolysaccharides (dEPS) were produced by our lab as we previously reported from Chaetoceros socialis (Norwegian Culture Collection strain K1676) (59, 111). ^13^C-labeled EPS from this organism was chosen because it is an ecologically relevant phytoplankton species and has a wide geographic distribution. Since sinking diatom biomass is a major contributor to the organic carbon content of Namibian shelf sediments (110), the ^13^C-labeled OM representing a mixture of dead diatom cells and their EPS serves as an appropriate proxy for tracking activity of heterotrophic microbes in the BUS.

*C. socialis* cells were grown in 250 mL sterile polystyrene culture flasks (VWR International) with 1 L growth medium (114) at 22°C for 7 days exposed to the natural light-dark cycle (flasks were placed in an east-facing window). One set of cultures was grown with 2 mM 99% ^13^C-labeled sodium bicarbonate, and another set was grown with unlabeled sodium bicarbonate. After 7 days the cultures were turbid, as evidenced by mucosal light brown flocculants (EPS and colonies of diatom cells); the cultures at that point were centrifuged, the particulate fraction from the concentrate was collected as a pellet, the supernatant was removed, and the pellets were dried in a sterile laminar flow hood prior to the cruise. Thus, the resulting cell culture concentrates consisted of particulate OM that was used as an inoculum for the stable isotope probing (SIP) experiments. Gas chromatography isotope mass ratio spectrometry (GC-IRMS) was used to determine that the atom percent ^13^C enrichment of the OM was >50% (60). Prior to the cruise, the cell culture concentrates were treated twice with a DNase enzyme (Turbo DNase; Life Sciences), increasing the incubation times to 1 h, in order to remove ^13^C-labeled DNA from the diatoms that may otherwise have accumulated in the heavy fractions of the CsCl gradient postincubation and thus potentially biased comparisons of functional gene relative abundances in the heavier SIP fractions.

For seawater ^13^C-SIP incubations, water from the Niskin rosettes at 10 mwd and 125 mwd at site 6 was sampled immediately on board the ship in 1-L borosilicate glass flasks (Duran; DWK Life Sciences GmbH). Bottles received either the unlabeled (control) or ^13^C-labeled dEPS at a final concentration of 0.2 mg · L^−1^. For each incubation with dEPS, 1 L of seawater was transferred to glass flasks with the added substrate and capped, leaving no air in the headspace. Bottles were incubated in the dark at 10°C for 18 h, with continuous monitoring of O_2_ concentrations with a noninvasive fiber optic method, as described previously (115). At the end of the incubation, the seawater was filtered onto 0.2-μm filters (Pall Supor-200) using a peristaltic pump that were then immediately stored in sterile, DNA/RNA clean 15-mL Falcon tubes and flash frozen at −80°C. DNA was extracted from the filters as described above using a previously published protocol (60).

Core sediments from 28 cm below the seafloor (cmbsf) at site 6 were selected for ^13^C-SIP incubations. In the 28-cmbsf SIP incubations with ^13^C-labeled sodium bicarbonate, sediment was added to 20-mL glass flasks leaving no headspace (ca. 20 g sediment) that were crimp-sealed using gray butyl rubber stoppers. Samples were incubated inside sterile glass bottles and supplemented with ^13^C-labeled bicarbonate (and bicarbonate as a control) at a concentration of 2 mM in triplicate for 10 days at 10°C in the dark. Flasks received either 2 mM 99% ^13^C-labeled or unlabeled (control) sodium bicarbonate (NaHCO_3_; Sigma-Aldrich). At the end of the 10-day incubation, bottles were flash frozen at −80°C. DNA was extracted from the sediment as described above. Sediment SIP incubations were extended for 10 days (instead of 18 h like the water column), because they were strictly anoxic, and we expected rates of microbial activity (and ^13^C-assimilation) to be slower as a result.

We processed DNA extracts for density gradient centrifugation according to published protocols (61, 116, 117). DNA from the 15 pools fractionated via density gradient was eluted into 30 μL molecular-grade water (Roche) and quantified using a Qubit 3.0 fluorometer. To determine shifts in the peak buoyant density of DNA, qPCR assays targeting the V4 hypervariable region of 16S rRNA genes were carried out on the 15 density fractions as described hereafter. The ^13^C- labeled fractions with the highest gene copy numbers were selected and pooled for metagenomic library preparation.

### 16S rRNA gene quantification.

DNA templates were used in qPCR amplifications with updated 16S rRNA gene primer pair 515F (5′- GTG YCA GCM GCC GCG GTA A-3′) with 806R (5′-GGA CTA CNV GGG TWT CTA AT-3′) to increase our coverage of *Archaea* and marine clades (118) and run as previously described (117, 119). The reaction efficiencies in all qPCR assays were between 90% and 110%, with an r^2^ of 0.98. Gene copies were normalized to the wet weight of the sediment and volume of water filtered.

### Library preparation.

**(i) 16S rRNA genes.** 16S rRNA gene amplicons were run on 1.5% agarose gels, the bands were excised and purified with the QIAquick gel extraction kit (Qiagen), and the final eluted DNA was quantified with the Qubit double-stranded DNA (dsDNA) high-sensitivity (HS) assay kit (Thermo Fisher Scientific). Purified PCR amplicons containing unique barcodes from each sample were diluted to 1 nM solutions and pooled. Library preparation was carried out according to the MiniSeq System Denature and Dilute Libraries Guide from Illumina, and a 500-μL library (1.8 pM) with 8 μL denatured PhiX control were sequenced on the Illumina MiniSeq platform using an Illumina MiniSeq midoutput kit (300-cycles), as described previously (120).

**(ii) Metagenomes and metatranscriptomes.** Initial DNA extracts were diluted to DNA concentrations of 0.2 ng μL^−1^ and used in metagenomic library preparations with the Nextera XT DNA library prep kit (Illumina). Then, 10 μL of RNA templates was processed for DNase treatment and synthesis of cDNA and library construction with specific barcodes using the Trio RNA-Seq kit protocol (NuGEN Technologies). Because the Trio RNAseq Ovation kit (NuGen Technologies) is biased against molecules with secondary structure such as rRNA and preferentially amplifies mRNA, we did not perform an rRNA depletion step. Metagenomic and metatranscriptomic libraries were quantified on an Agilent 2100 bioanalyzer system, using the high-sensitivity DNA reagents and DNA chips (all Agilent Genomics), and diluted to 1 nM. For further sequencing, we pooled up to five libraries and sequenced them in two separate runs with a paired-end 300 midoutput kit on the Illumina MiniSeq platform (120).

### Assembly and analysis.

**(i) 16S rRNA genes.** We performed demultiplexing and base calling using bcl2fastq conversion software v. 2.18 (Illumina). We used USEARCH (121) for MiniSeq read trimming and assembly, OTU picking, and clustering at 97% sequence identity. Taxonomic assignments were generated by QIIME, v. 1.9.1 (122), using the implemented BLAST method against the SILVA rRNA gene database, release 138 (123). We removed all OTUs containing <10 sequences and those which had no BLASTn hit. Reads passing quality control were normalized by the percentage of total sequencing depth per sample. The SqueezeMeta metagenomic analysis pipeline (124) was used to predict and extract 16S rRNA genes identified in both metatranscriptomic and SIP metagenomic libraries, using Barnnap coassembly mode (125). The corresponding sequences were processed for phylogenetic analysis as described above.

**(ii) Metagenomes, metatranscriptomes.** Quality control, *de novo* assembly, and open reading frame (ORFs) searches were performed as described previously (60, 115), with some minor modifications. The MiniSeq reads were trimmed, and paired-end reads were assembled into contigs, using CLC Genomics Workbench v. 9.5.4 (https://www.qiagenbioinformatics.com/), using a word size of 20, bubble size of 50, and a minimum contig length of 300 nucleotides. Reads were mapped to the contigs using the following parameters: mismatch penalty = 3, insertion penalty = 3, deletion penalty = 3, minimum alignment length = 50% of read length, minimum percent identity = 95%. Coverage values were obtained from the number of reads mapped to a contig divided by its length (i.e., average coverage). Only contigs with an average coverage of >5 were selected for ORF searches and downstream analysis. This protocol does not assemble rRNA, and thus transcript results are only discussed in terms of mRNA. Protein-encoding genes and ORFs were extracted using FragGeneScan v. 1.30 (126).

To assign taxonomic affiliation to metagenomic and metatranscriptomic data (127), we applied our previously published bioinformatics pipeline (128) that involves a large aggregated genome database of predicted proteins, including the SEED (www.theseed.org) and NCBI RefSeq (www.ncbi.nlm.nih.gov/) databases updated with all predicted proteins from recently described high-quality draft metagenome-assembled genomes (MAGs) and single-cell-assembled genomes (SAGs) from the NCBI protein database. Taxonomic identifications were integrated with functional annotations by performing BLASTp and BLASTx searches of ORFs against our aggregated database of predicted proteins. We used the DIAMOND protein aligner v. 0.9.24 (129). Cutoff values for assigning the best hit to specific taxa were performed at a minimum bit score of 50, a minimum amino acid similarity of 60, and an alignment length of 50 residues. We use this approach to draw conclusions about metabolic traits derived specifically from higher-level taxonomic groups only (60, 106). We chose to focus on the coverage of total annotated protein-encoding ORFs detected, as opposed to the number of reads mapping per kilobase per ORF (for example, reads per kilobase per million [RPKM]), to reduce potential bias from small numbers of “housekeeping” genes with potentially higher expression levels (128).

For 16S rRNA gene amplicon data sets, each sample was sequenced to an average depth of 22,876 sequences per sample (standard deviation = 3,871) via Illumina sequencing. Metatranscriptomes spanning water column and seafloor habitats (*n* = 27) were sequenced with an average depth of 5.9 million reads (standard deviation = 1.5), and after *de novo* assembly an average of 17,943 contigs per sample could be assembled (standard deviation = 7,216) (Table S1). Metagenomes were sequenced to an average depth of 6.3 million reads per sample (standard deviation = 1.7) (Table S1). For the heavy metagenomes prepared from the labeled SIP fractions, the SIP metagenomes were sequenced to an average depth of 6.6 million reads (standard deviation = 2.2) (Table S1). Statistical analyses of beta diversity were performed using RStudio v. 3.3.3 with the Bioconductor package (130). The Anvi’o snakemake workflow (131, 132) was used for downstream binning analysis, using coassembly mode with default settings (Table S2).

### Phylogeny of functional genes.

Phylogenetic analyses of the predicted alpha subunits of the dissimilatory sulfite reductase (*dsrA*) and adenylylsulfate reductase (*aprA*) gene proteins were performed for all the corresponding annotated taxa in our metagenomes and metatranscriptomes, using 466 and 433 aligned amino acid sites, respectively (82). For each of the two marker gene phylogenies (*dsrA*, *aprA*), all ORFs annotated to those genes from our bioinformatics pipeline were aligned against their top two BLASTp hits in the NCBI nonredundant (nr) and SEED databases using MUSCLE (133). Conserved regions of the alignments were selected in SeaView v. 4.7 (134), using Gblocks with the following settings: allowing for smaller final blocks, gap positions within the final blocks, and less strict flanking positions. Phylogenetic analyses of the resulting amino acid alignments of the predicted proteins were conducted in SeaView v. 4.7 (134) using RAxML (135) with BLOSUM62 as the evolutionary model and 100 bootstrap replicates. We processed phylogenetic analyses of TonB-dependent transporters (*TonB*) and citrate transport proteins (*citN*) for all the corresponding annotated ORFs in our SIP metagenomes, as described above using 1,233 and 480 aligned amino acid sites, respectively.

All 16S rRNA gene sequences extracted using SqueezeMeta (124) were identified by BLASTn searches against the SILVA 16S rRNA SSU NR99 reference database release 138 (123). All OTUs were aligned with SINA online v. 1.2.11 (136) and inserted in one Maximum Likelihood RAxML phylogenetic tree based on the closest BLASTn sequence matches, selecting the best tree among 100 replicates using ARB (137). Partial OTU sequences were added to the tree using the maximum parsimony algorithm without allowing changes of tree typology.

### Data availability.

All scripts and codes used to produce sequence analyses have been posted on GitHub with a link to the instructions for how to conduct the scripts (github.com/williamorsi/MetaProt-database). All metagenome, metatranscriptome, and 16S rRNA gene data are publicly accessible in NCBI through BioProject number PRJNA525353. All alignments for each of the four marker gene phylogenies (*dsrA*, *aprA*, *TonB*, *citN*) are publicly available through the LMU Open Data website (https://doi.org/10.5282/ubm/data.190).
