# Supplementary material for: Microbial Activities and Selection from Surface Ocean to Subseafloor on the Namibian Continental Shelf
Source: Appl Environ Microbiol. 2022 Apr 11;88(9):e00216-22. doi: 10.1128/aem.00216-22 (PMC9088280; doi:10.1128/aem.00216-22)
Supplement: Supplemental file 1 — Fig. S1 to S5 and Tables S1 and S2. Download aem.00216-22-s0001.pdf, PDF file, 5 MB [file aem.00216-22-s0001.pdf]

Supplemental Material

Microbial activities and selection from surface ocean to subseafloor on the Namibian continental shelf

Aurèle Vuillemin*, Ömer K. Coskun, and William D. Orsi

Content

Supplementary Figure S1. Sediment cores retrieved from the Namibian shelf.

Supplementary Figure S2. Geochemical profiles for the water column and sediment at site 6.

Supplementary Figure S3. Depth profiles of latitudinal gradients in chlorophyll-a fluorescence and turbidity.

Supplementary Figure S4. Metabolic functions and activities related to hydrogen in the water column, sediment and SIP incubations, and the corresponding taxonomic assignments at the phylum level.

Supplementary Figure S5. Phylogenetic analysis of ORFs encoding TonB-dependent and citrate transporters detected in SIP metagenomes.

Supplementary Table S1. Sequencing and assembly statistics for the metatranscriptomes, metagenomes, and SIP metagenomes.

Supplementary Table S2. Binning statistics for the metatranscriptomes.

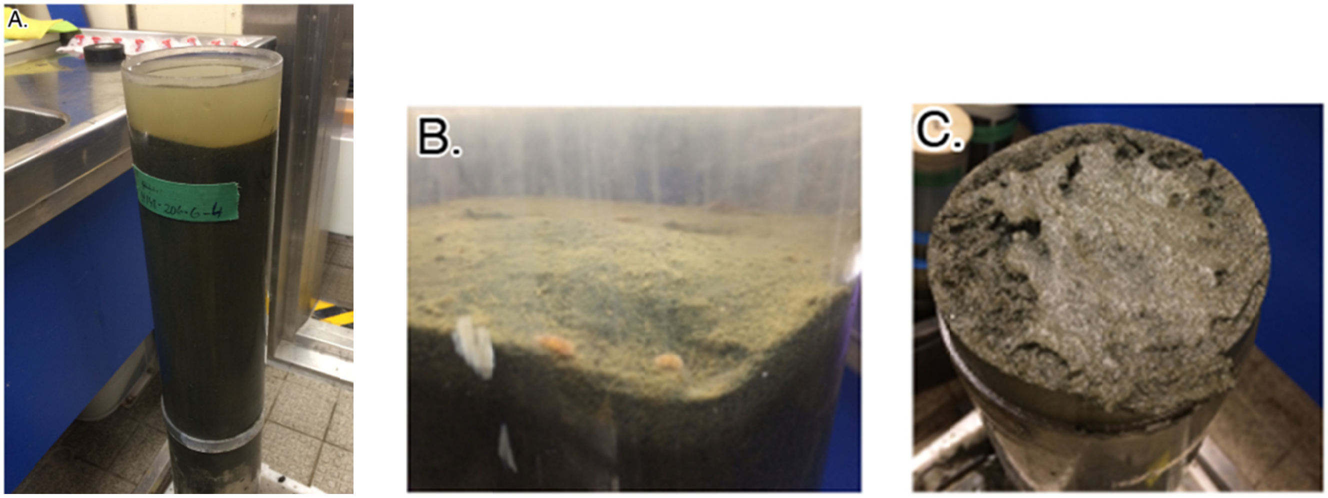

Supplementary Figure S1. Sediment cores retrieved from the Namibian shelf. (**A**) Pristine core recovered, together with bottom water. (**B**) Zoom in on the core top surface. (**C**) Photo of core inside at ca. 15 cm depth, taken during sectioning. The sediment is composed primarily of green mud and foraminiferal sand (99).

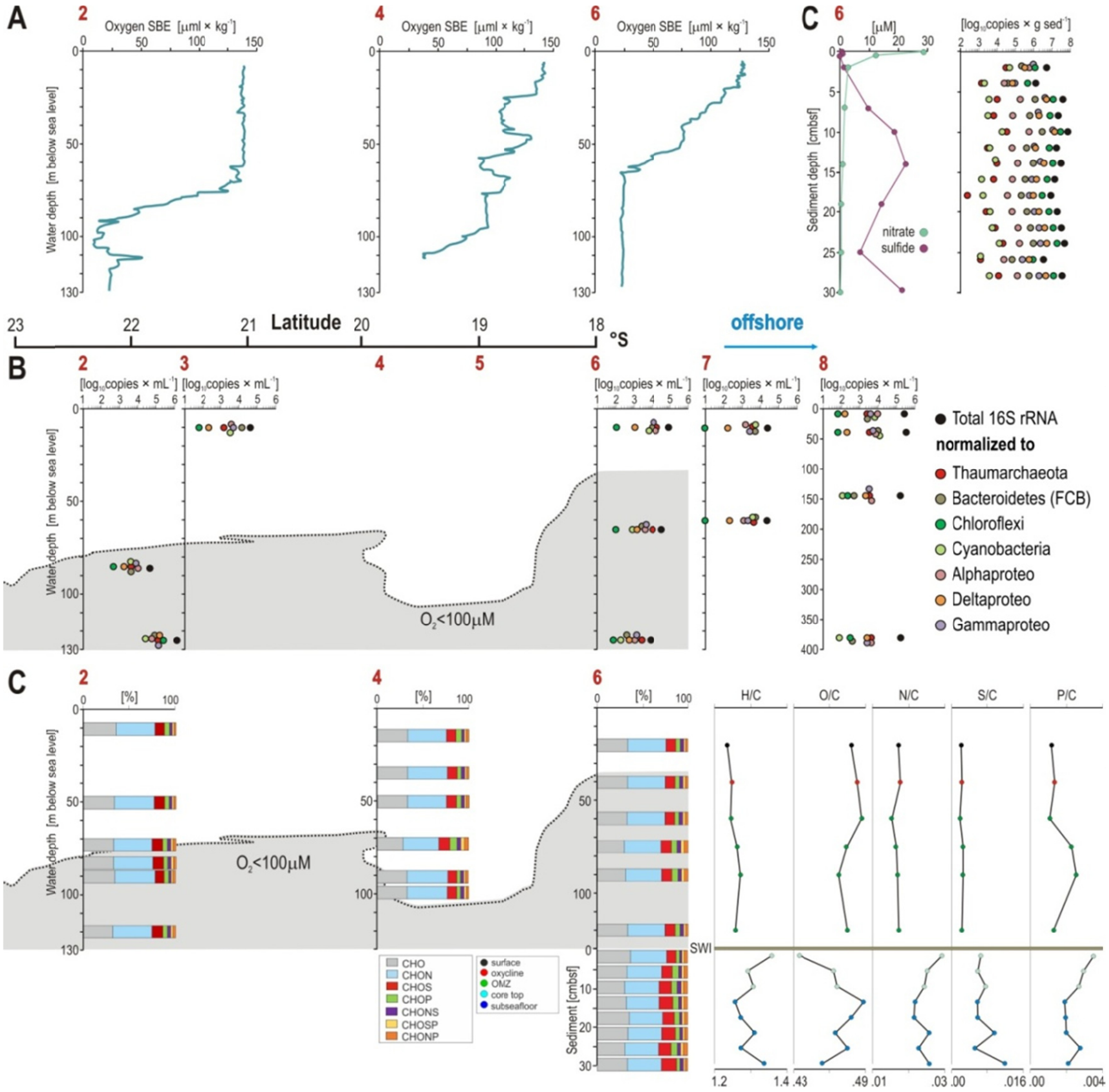

Supplementary Figure S2. Geochemical profiles for the water column and sediment and quantification of 16S rRNA genes normalized to specific phyla. (A) Vertical profile of dissolved O_2 in the water column at sampling site 2, 4 and 6 measured with the CTD. **(B)** Quantitative PCR (qPCR) of total 16S rRNA genes (black dots) in the water column and 16S rRNA genes normalized to the relative abundance of Thaumarchaeota (red), Bacteroidetes (brown), Chloroflexi (dark green), Cyanobacteria (light green), Alphaproteobacteria (pink), Deltaproteobacteria (orange), Gammaproteobacteria (mallow). **(C)** Geochemical profiles of nitrate (green) and sulfide (purple) showing a redox transition zone between 8 and 12 cmbsf. The sulfide data represent average measurements from two cores; and qPCR profiles for total (black dots) and same normalized 16S rRNA genes as above in the sediment 30 cmbsf. **(D)** Relative proportion of dissolved organic matter (DOM) categories (48) in the water column and sediments (measured via Fourier Transform ICR-Mass Spectrometry), vertical distribution of the DOM different molar ratios (H/C, O/C, N/C, S/C, P/C), showing that diffusion across the sediment-water interface was not active at the time of sampling.

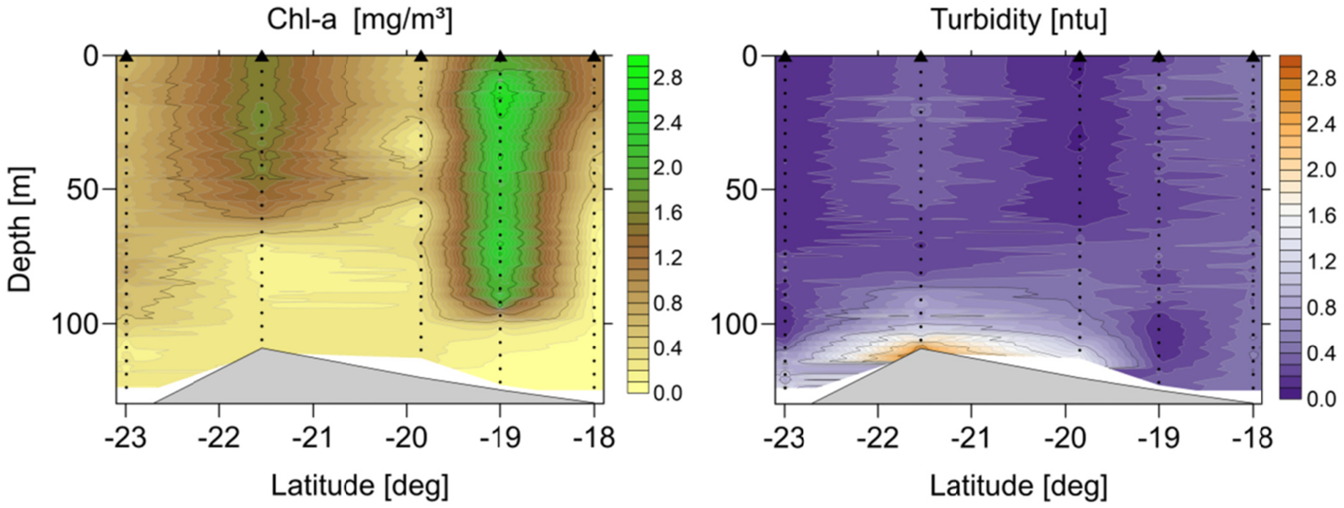

Supplementary Figure S3. Distribution of chlorophyll-a fluorescence and turbidity along the Northern Benguela Shelf transect. (**Left**) Chlorophyll-a fluorescence [mg × m^3], and (**right**) turbidity in nephelometric turbidity units [ntu] based on preliminary data of the EreBUS oceanographic cruise. The corresponding short cruise report is available at URL: https://www.ldf.uni-hamburg.de/en/meteor/wochenberichte/wochenberichte-meteor/m145-m148-2/m148-2-wob2e.pdf

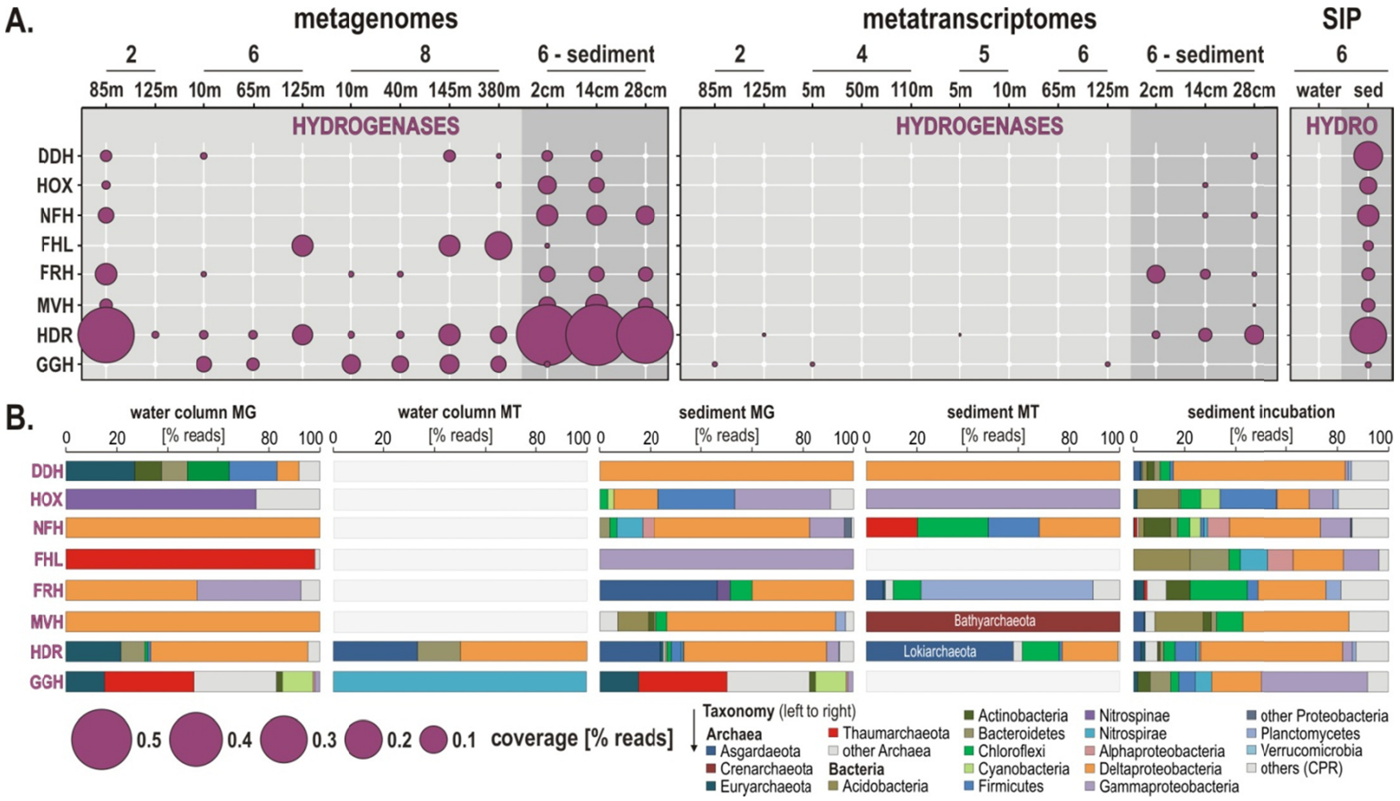

Supplementary Figure S4. Metabolic functions and activities related to hydrogen in the water column, sediment and SIP incubations, and the corresponding taxonomic assignments at the phylum level. (A) Bubble plot showing the relative potential and expression of metabolic functions [% reads] assigned to hydrogenases in the metagenomes, metatranscriptomes, and stable isotope probing (SIP) water and sediment incubations (left to right). (**B**) Taxonomic bar charts [% reads] for marker genes related to hydrogenases at the phylum level in the metagenomes (MG), metatranscriptomes (MT) and anaerobic SIP incubations with sediment and ^{13}C-labeled bicarbonate. Abbreviations: DDH: Fe-Fe hydrogenase / HOX: NAD-reducing hydrogenase / NFH: Ni-Fe hydrogenase / FHL: formate-hydrogen lyase / FRH: coenzyme F420 reducing and non-reducing hydrogenase / MVH: methyl-viologen hydrogenase / HDR: heterodisulfide reductase / GGH: geranylgeranyl hydrogenase.

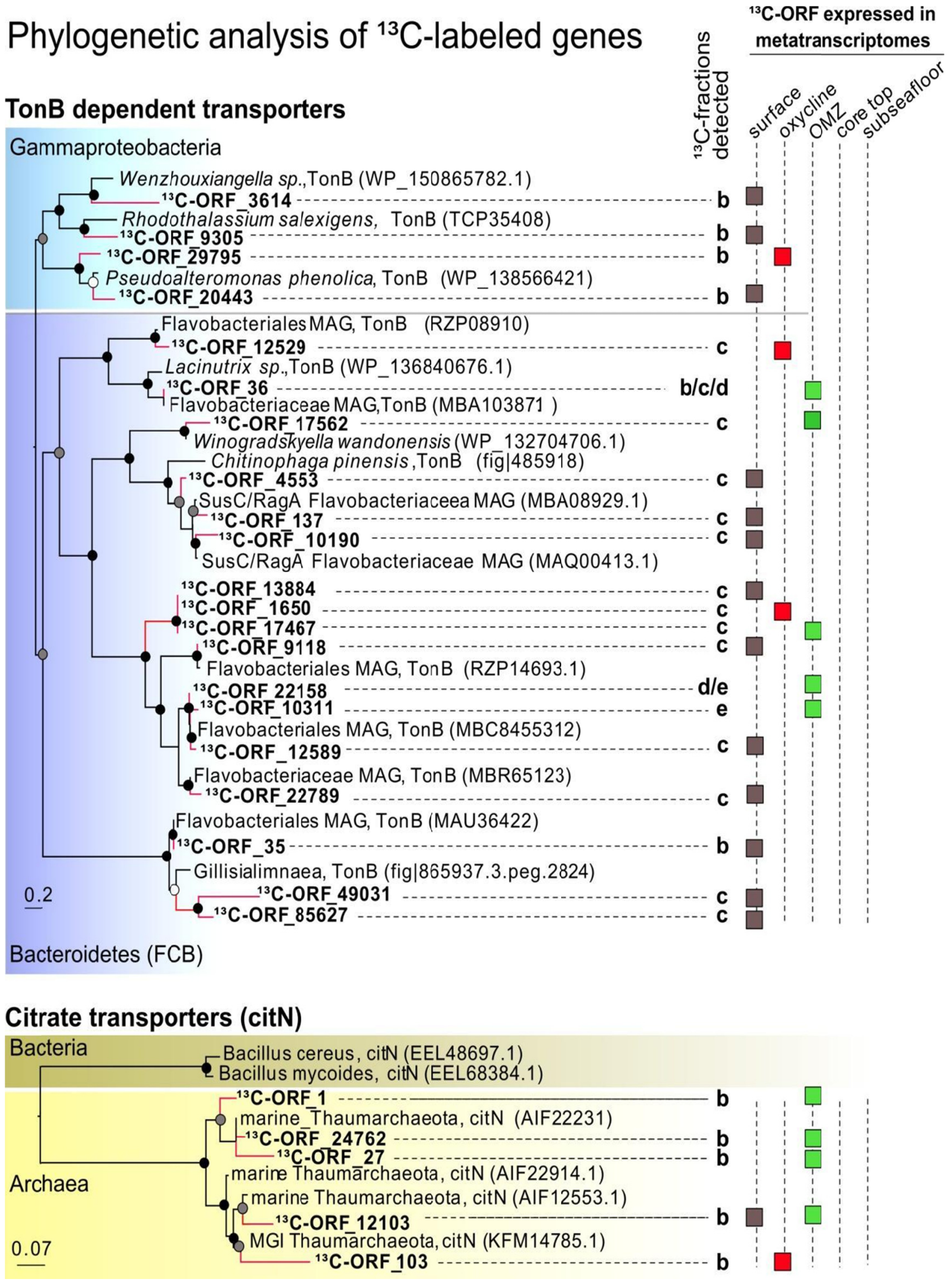

Supplementary Figure S5. Phylogenetic analysis of predicted proteins encoded by selected marker genes in the SIP metagenomes, based on RAxML using BLOSUM62 as the evolutionary model. (**A**) Phylogenetic tree of all TonB-dependent transporters (*TonB*) ORFs detected in the "heavy" ^{13}C-labeled DNA fractions (a to e) of the SIP metagenomes (1,233 aligned amino acid sites). (**B**) Phylogenetic tree of all citrate transporters (*citN*) ORFs detected in the SIP metagenomes (480 aligned amino acid sites).

Supplementary Table S1. Sequencing and assembly statistics for metatranscriptomes, metagenomes, and SIP metagenomes.

METATRANSCRIPTOMES

Station	Sample type	oxygen level	Depth (m)	Replicate	# reads (millions)	# contigs	reads mapping (millions)	% of reads mapping to contigs
site-202	water column	oxycline	85	a	3	13,274	2.1	70.0
				b	3.6	27,862	2.2	61.1
		OMZ	125	a	4.7	19,254	3.5	74.5
				b	3.4	15,817	2.5	73.5
site-204	water column	Surface (oxic)	5	a	10.2	67,010	8.2	80.4
				b	9.1	41,408	7	76.9
		oxycline	50	a	9.4	21,960	8.2	87.2
				b	7.6	17,647	6.6	86.8
		OMZ	110	a	13.8	27,168	12.2	88.4
				b	7.9	29,864	6.2	78.5
site-206	water column	oxic	5	a	5.7	19,334	4.6	80.7
				b	6.7	37,547	5.1	76.1
			10	a	7.2	20,735	6.3	87.5
				b	3.7	10,464	3.2	86.5
		oxycline	65	a	7.2	16,298	6.1	84.7
				b	3.5	9,860	2.7	77.1
		OMZ	125	a	1.7	5,526	1.3	76.5
				b	5.3	16,420	4.2	79.2
				c	7.7	25,788	6.1	79.2
	sediments	hypoxic	core top	a	4.6	2,602	3.7	80.4
				b	11.1	2,927	9.2	82.9
		sulfidic	12 cm	a	3.8	4,362	2.7	71.1
				b	2.2	2,726	1.3	59.1
				c	3.4	5,888	2.1	61.8
			28 cm	a	3.8	7,429	2.3	60.5
				b	5.7	9,636	4.2	73.7
				c	4.1	5,660	2.7	65.9

METAGENOMES

Station	Sample type	oxygen level	Depth (m)	Replicate	# reads (millions)	# contigs	reads mapping (millions)	% of reads mapping to contigs
site-202	water column	oxycline	85	a	5.5	79,040	1.7	30.9
		OMZ	125	a	3.6	81,412	0.6	16.7
site-206	water column	oxic	10	a	4.9	102,742	1.5	30.6
		oxycline	65	a	5.8	95,018	1.2	20.7
		OMZ	125	a	7.1	49,885	0.88	12.4
	sediments	hypoxic	core top	a	15.7	80,933	1.26	8.0
		sulfidic	12 cm	a	5.1	12,580	0.15	2.9
			28 cm	a	3.6	9,583	0.12	3.3
site-208	water column	oxic	10	a	5	70,549	1.2	24.0
			145	a	7.8	125,658	1.9	24.4
			380	a	5	79,761	1.4	28.0

SIP-metagenomes

Station	SIP incubation	Sample type	oxygen level	Depth (m)	^{13}C-SIP metagenome	Density range (g mL^{-1})	# reads (millions)
site-206	^{13}C-dEPS	water column	oxic	10m	10m_a	1.685-1.70	4.9
					10m_b	1.70-1.74	4.7
					10m_c	1.74-1.76	3.7
			OMZ	125m	125m_a	1.70-1.73	5.8
					125m_b	1.73-1.745	2.7
	^{13}C-bicarbonate	sediment	sediment	23 cm	23cm_a	1.682	9.6
					23cm_b	1.689	2.7
					23cm_c	1.697	15.5
					23cm_d	1.705	12.8
					23cm_e	1.713	8.7
					23cm_f	1.722	1.6

Supplementary Table S2. Binning statistics for metatranscriptomes.

Sample	total_num_reads	num_INDELs_reported	total_reads_kept	num_SNVs_reported	total_reads_mapped	percent_mapped
Site 202 85m (MT)	5994376	7390	2543311	163023	2543311	42.43
Site 202 125m (MT)	6646454	10235	3257803	245540	3257803	49.02
Site 204 5m (MT)	17164574	12126	8786590	259866	8786590	51.19
Site 204 50m (MT)	15809144	13804	8992028	321241	8992028	56.88
Site 204 110m (MT)	21100648	9460	15912601	161755	15912601	75.41
Site 206 5m (MT)	11629756	8151	5088200	139498	5088200	43.75
Site 206 10m (MT)	11399926	14046	5529901	320131	5529901	48.51
Site 206 65m (MT)	9887270	11220	5402852	249057	5402852	54.64
Site 206 125m (MT)	20230136	13643	10912948	344857	10912948	53.94
Site 206 core top3 (MT)	3393648	7991	1706512	111746	1706512	50.29
Site 206 core top (MT)	9222134	12171	4416491	172450	4416491	47.89
Site 206 core 12cm (MT)	8446572	9040	2695838	213050	2695838	31.92
Site 206 28cm (MT)	59101508	17092	6830510	943736	6830510	11.56

bins	total_length	num_contigs	N50	GC_content	percent_completion	percent_redundancy	t_domain	t_phylum	t_class	t_order	t_family	t_genus	t_species
MAXBIN__031	6449290	3961	1549	52.0	91.55	67.61	Bacteria	Desulfobacterota	Syntrophobacteria	BM002	BM002	BM002	BM002 sp002899795
MAXBIN__034	6583881	4089	1513	63.6	87.32	88.73	Bacteria	Actinobacteriota	Acidimicrobiia	UBA5794	UBA4744	UBA4744	UBA4744 sp002403855
MAXBIN__036	3542706	2068	1711	64.1	67.61	32.39	Bacteria	Myxococcota					
MAXBIN__033	2915624	1980	1428	61.7	63.38	35.21	Bacteria	Myxococcota	Polyangia	Polyangiales	SG8-38	SG8-38	SG8-38 sp003647035
MAXBIN__019	738826	497	1426	47.0	60.56	92.96	Bacteria						
MAXBIN__003	2410097	1526	1558	45.3	54.93	21.13	Bacteria	Proteobacteria	Gammaproteobacteria	Enterobacterales	Alteromonadaceae	Pseudoalteromonas	
MAXBIN__009	434046	258	1616	41.1	50.70	53.52	Bacteria	Proteobacteria	Gammaproteobacteria	PS1	Thioglobaceae	Thioglobus	Thioglobus singularis
MAXBIN__018	102837	66	1513	32.6	50.70	36.62	Bacteria	Bacteroidota	Bacteroidia	Flavobacteriales	Flavobacteriaceae	MAG-121220-bin8	MAG-121220-bin8 sp004214185
MAXBIN__032	2237305	1428	1537	46.9	47.89	11.27	Bacteria	Desulfobacterota	Syntrophobacteria	BM002	BM002	BM002	BM002 sp002899795
MAXBIN__025	489338	327	1400	30.7	47.89	43.66	Bacteria	Bacteroidota	Bacteroidia	Flavobacteriales			
MAXBIN__035	2493781	1879	1276	69.4	42.25	19.72	Bacteria	Myxococcota	UBA9160	UBA9160	UBA6930	UBA6930	UBA6930 sp002450755
MAXBIN__024	233551	157	1386	43.5	42.25	23.94	Bacteria						
MAXBIN__007	267590	156	1683	32.2	39.44	4.23							
MAXBIN__021	1617878	1099	1373	39.7	38.03	35.21	Bacteria	Proteobacteria	Gammaproteobacteria	PS1	Thioglobaceae	Thioglobus	Thioglobus sp001628405
MAXBIN__026	1308179	908	1368	42.4	36.62	22.54							
MAXBIN__011	106535	48	2371	45.0	32.39	4.23	Bacteria	Proteobacteria	Gammaproteobacteria	Pseudomonadales	Porticoccaceae	HTCC2207	
MAXBIN__013	944322	704	1286	49.2	30.99	11.27	Bacteria	Proteobacteria	Gammaproteobacteria	Pseudomonadales			
MAXBIN__016	222421	166	1293	37.2	29.58	22.54	Bacteria	SAR324	SAR324	SAR324	NAC60-12	Arctic96AD-7	Arctic96AD-7 sp002685535
MAXBIN__022	133005	86	1570	38.4	28.17	19.72	Bacteria						
MAXBIN__014	177509	132	1304	32.9	26.76	28.17	Bacteria	Bacteroidota	Bacteroidia	Flavobacteriales	Flavobacteriaceae	MAG-121220-bin8	MAG-121220-bin8 sp002700465
MAXBIN__023	307720	215	1307	37.5	23.94	14.08	Bacteria						
MAXBIN__015	102878	67	1501	41.1	23.94	4.23	Bacteria	Verrucomicrobiota	Lentisphaeria	Lentisphaerales	Lentisphaeraceae	Lentisphaera	Lentisphaera araneosa
MAXBIN__038	1660543	1282	1246	67.7	22.54	9.86							
MAXBIN__010	993817	673	1409	40.5	22.54	21.13	Bacteria	Proteobacteria	Gammaproteobacteria	PS1	Thioglobaceae	Thioglobus	Thioglobus singularis
MAXBIN__006	838719	546	1501	41.4	22.54	8.45	Bacteria						
MAXBIN__004	123978	69	1799	35.6	21.13	1.41	Bacteria	Marinisomatota	Marinisomatia	Marinisomatales	TCS55	TCS55	TCS55 sp001577025
MAXBIN__037	1869842	1462	1221	66.9	0.00	0.00							
MAXBIN__008	1481191	950	1536	42.0	0.00	0.00							
MAXBIN__029	733196	440	1500	35.9	0.00	0.00	Bacteria						
MAXBIN__002	668219	440	1498	46.9	0.00	0.00	Bacteria	Proteobacteria	Gammaproteobacteria	Enterobacterales	Vibrionaceae	Aliivibrio	Aliivibrio salmonicida
MAXBIN__028	635327	444	1369	47.3	0.00	0.00	Bacteria						
MAXBIN__017	499776	310	1538	35.0	0.00	0.00	Bacteria	Bacteroidota	Bacteroidia	Flavobacteriales	BACL11	UBA8444	UBA8444 sp003454845
MAXBIN__001	248620	152	1585	46.4	0.00	0.00							
MAXBIN__012	239179	165	1417	31.5	0.00	0.00							
MAXBIN__005	207317	135	1573	36.4	0.00	0.00	Bacteria	Proteobacteria	Gammaproteobacteria	PS1	Thioglobaceae	Thioglobus	Thioglobus singularis
MAXBIN__027	155301	122	1174	34.0	0.00	0.00							
MAXBIN__020	121882	81	1527	31.4	0.00	0.00	Bacteria	Bacteroidota	Bacteroidia	Flavobacteriales	Flavobacteriaceae	MED-G11	
MAXBIN__030	102295	53	1953	33.1	0.00	0.00	Bacteria	Bacteroidota	Bacteroidia	Flavobacteriales	Flavobacteriaceae	Maribacter	Maribacter sp000153165
